# Comparing the Accuracy of Different Wearable Activity Monitors in Patients With Lung Cancer and Providing Initial Recommendations: Protocol for a Pilot Validation Study

**DOI:** 10.2196/70472

**Published:** 2025-06-19

**Authors:** Roberto M Benzo, Rujul Singh, Carolyn J Presley, Macy K Tetrick, Zachary L Chaplow, Chloe M Hery, Jane Yu, Peter Washington, Frank J Penedo, Electra D Paskett, Vipul Lugade, Emma Fortune

**Affiliations:** 1 Division of Cancer Prevention and Control, Department of Internal Medicine, The Ohio State Comprehensive Cancer Center College of Medicine The Ohio State University Wexner Medical Center Columbus, OH United States; 2 Division of Medical Oncology, Department of Internal Medicine, The Ohio State Comprehensive Cancer Center College of Medicine The Ohio State University Wexner Medical Center Columbus, OH United States; 3 Kinesiology Department of Human Sciences The Ohio State University Columbus, OH United States; 4 College of Medicine The Ohio State University Columbus, OH United States; 5 Division of Clinical Informatics and Digital Transformation Department of Medicine University of California - San Francisco San Francisco, CA United States; 6 Departments of Psychology and Medicine University of Miami Coral Gables, FL United States; 7 Sylvester Comprehensive Cancer Center Miller School of Medicine University of Miami Miami, FL United States; 8 Motion Analysis Research Laboratory The State University of New York Binghamton Binghamton, NY United States; 9 Division of Health Care Delivery Research Mayo Clinic Rochester, MN United States; 10 Robert D and Patricia E Kern Center for the Science of Health Care Delivery Mayo Clinic Rochester, MN United States

**Keywords:** physical activity monitoring, wearable devices, validation study, lung cancer, consumer technology, Fitbit, ActiGraph, activPAL3 micro, cancer survivorship, digital health

## Abstract

**Background:**

Wearable activity monitors (WAMs) provide insights into physical activity (PA) and are widely used in behavioral interventions and cancer survivorship research. However, validation studies of wearable devices in populations with cancer are scarce, and existing studies using activity monitors in patients with cancer lack standardization. This gap is particularly significant in patients with lung cancer (LC), who often experience unique mobility challenges and gait impairments that may affect device accuracy. This study addresses this gap by validating the Fitbit Charge 6, ActiGraph LEAP, and activPAL3 micro in patients with LC in both laboratory and free-living conditions and developing a standardized framework for assessing wearable devices in populations with cancer and impaired mobility.

**Objective:**

This study aims to validate and compare the accuracy of consumer-grade (Fitbit Charge 6) and research-grade (activPAL3 micro and ActiGraph LEAP) WAMs in patients with LC under both laboratory and free-living conditions. Moreover, this protocol aims to establish standardized procedures that can be adapted for validating current and future generations of wearable devices while accounting for disease-specific factors that may impact measurement accuracy.

**Methods:**

In total, 15 adults diagnosed with LC (stages 1-4) will participate in laboratory and free-living protocols, wearing Fitbit Charge 6, activPAL3 micro, and ActiGraph LEAP devices simultaneously. The laboratory protocol will consist of a series of structured activities, including variable-time walking trials, sitting and standing tests, posture changes, and gait speed assessments. Activities will be video recorded for validation. In the free-living protocol, participants will wear the devices continuously for 7 days except during water-based activities. WAM-based outcome measures will include step count; time spent at light, moderate, and vigorous PA intensity levels; posture; and posture changes (only the activPAL3 micro measured posture). Validated survey instruments will be administered both before and after WAM data collection to control for potential confounding factors that may influence movement patterns and device accuracy. Laboratory-based validity measures will compare WAM data to video-recorded observations. Sensitivity, specificity, positive predictive value, and agreement will also be determined. Free-living agreement between devices will be assessed using Bland-Altman plots, intraclass correlation analysis, and 95% limits of agreement.

**Results:**

Data collection is ongoing, with 11 participants enrolled and 7 (64%) having completed both in-laboratory and free-living protocols. On average, enrolled participants are aged 63.0 (SD 7.8; range 50.0-73.0) years, with 8 (73%) participants being women. Participant enrollment is expected to conclude in mid-2025, and initial findings are expected to be disseminated by the end of 2025.

**Conclusions:**

This is the first study that validates WAM accuracy for populations with LC while providing comprehensive recommendations for future validation studies. This study will provide critical insights into the accuracy and reliability of WAMs for assessing PA in LC survivors, which are essential for interpreting clinical research and informing future interventions.

**International Registered Report Identifier (IRRID):**

DERR1-10.2196/70472

## Introduction

### Background

Lung cancer (LC) is the most diagnosed cancer worldwide (with approximately >2.5 million new cases in 2022) and the leading cause of death due to cancer (with an estimated 1.8 million deaths in 2022) [1]. In the United States alone, there are an estimated 384,000 LC survivors, and as early detection methods improve, that number is rapidly increasing [2]. Although treatments are quickly improving, patients still experience debilitating disease–related symptoms, such as cough, chest pain, fatigue, and depression. However, new studies have shown that moderate exercise during LC treatment can lead to significant improvements in fatigue, anxiety, stress, self-esteem, cardiovascular fitness, muscle strength, gastrointestinal side effects, breathing, cancer recurrence, and overall mortality [3,4].

In recent years, wearable devices, such as fitness trackers, watches, and mobile sensors, have become increasingly popular. User penetration rates of fitness trackers are expected to reach 13.4% by 2029, and fitness tracker–derived measurements are being incorporated more frequently into patient health records [5,6]. The well-established benefits of physical activity (PA) on cancer outcomes have prompted researchers and clinicians to explore new methods for monitoring and managing patient health. Consumer-grade and research-grade wearable devices are increasingly being investigated as tools to track PA, heart rate, and cardiorespiratory fitness in patients with cancer [7]. These devices offer the potential for more comprehensive and continuous health monitoring, which could lead to improved management strategies and better outcomes for individuals undergoing cancer treatment [8]. Wearable devices are also often used to implement (eg, process measures, feedback, and goal setting) and evaluate PA interventions [9]. Implementation feasibility is essential when considering wearable devices in cancer care. Miyaji et al [10] demonstrated high adherence rates (90% of the patients used wearable activity monitors [WAMs] as instructed for >70% of the observation period) among both patients with gastrointestinal cancer and patients with LC using commercially available WAMs over a 28-day study, confirming their clinical feasibility. Similarly, Gupta et al [11] reported high adherence rates (96% of the patients used WAMs as instructed for >50% of the observation period) among patients with cancer using WAMs over a 12-week longitudinal study. Consequently, assessing health behaviors, such as PA and sedentary behavior (SB), using wearable devices in an individual’s free-living environment provides widespread opportunities for public health–related research and clinical applications.

Wearable devices such as the consumer-grade Fitbit Charge (Google LLC) and the research-grade activPAL3 micro (PAL Technologies) and ActiGraph GT3X have been extensively studied and validated against criterion measures, such as indirect calorimetry, validated research-grade devices, direct observation (DO), video analysis, and polysomnography (ie, a diagnostic test for sleep disorders), in healthy adult populations [12-15]. However, unlike healthy populations, patients with cancer (and particularly patients with LC) frequently experience respiratory-, balance-, and mobility-related impairments that alter typical movement patterns and present unique challenges when conducting studies using activity monitor–derived outcome measures [16]. These challenges are particularly evident in gait speed, as patients with cancer often exhibit significantly slower walking velocities compared to healthy adults, and the accuracy of most activity monitors has been shown to decrease substantially at slower walking speeds [17]. Moreover, physiological and behavioral differences, cancer treatment side effects, adherence issues in wearing activity monitors, and environmental factors may all impact wearable devices’ accuracy and reliability.

### Objectives

Despite the increasing use of research-grade and consumer-grade wearable devices for PA tracking in patients with cancer and the known challenges with their use in this population, validation studies are scarce, and none have specifically examined device accuracy in patients with LC. Moreover, studies using wearable activity monitoring devices in populations with cancer show significant heterogeneity in their protocols [18,19], and there are a few published protocol papers to guide researchers toward standardization. This lack of standardized validation procedures makes it challenging to compare results across studies and establish evidence-based recommendations for device selection and implementation in clinical practice. Therefore, this paper aims to describe a comprehensive protocol for validating both research-grade and consumer-grade WAMs in patients with LC, incorporating both laboratory and free-living conditions to provide a more complete understanding of device performance in patients with LC. In addition, this paper provides recommendations and considerations for future PA validation studies of wearables in similarly affected populations.

## Methods

### Study Design

The validation protocol will contain (1) in-laboratory and (2) free-living environment components. The purpose of the in-laboratory protocol will be to assess the validity and reliability of 2 research-grade activity monitors, namely activPAL3 micro and ActiGraph LEAP, and 1 consumer-grade device, Fitbit Charge 6, by comparing them to the gold-standard DO [20]. In addition, the purpose of the free-living protocol will be to evaluate the validity and reliability of the Fitbit Charge 6 compared to the criterion measures from 2 research-grade devices (ActiGraph LEAP and activPAL3 micro).

A series of surveys and self-report assessments will be administered to evaluate potential confounding variables due to stress, health-related quality of life (HRQoL), PA, sleep, symptom burden, and user-reported acceptability of the devices being validated.

### Participants and Recruitment

On the basis of previous work, our target is to enroll 15 participants for the study [20,21]. Our sample size of 15 participants aligns with similar validation studies of activity monitors [22,23]. Despite this relatively small number of individuals, the study design generates a robust dataset through multiple observations per participant. Each participant contributes hundreds to thousands of daily steps, numerous walking trials, static posture tasks, and posture changes. In addition, each person provides 7 full days of free-living data (exceeding 100 h per person). Given the challenges in recruiting patients with LC, this approach creates a statistically efficient dataset that is adequate for validation purposes. Inclusion criteria are participants aged between 18 and 89 years; a diagnosis of stages 1-4 of LC; the ability to speak, read, and understand English; the ability to engage in PA; and having access to email. Exclusion criteria are pregnancy or plans to become pregnant and administration of adjunct therapy within 7 days of the baseline laboratory visit. Therapies, such as oral tyrosine kinase inhibitors (TKIs) and hormonal therapies will be permitted during the study period, given that both treatments are administered continuously and are very commonly administered treatments for patients with cancer [24,25]. The administration of adjunct therapy before or during the collection of activity monitor–derived data will be included as an exclusion criterion due to the high levels of fatigue experienced immediately after treatment in patients with cancer [26].

The recruitment process began in May 2024, and enrollment is still ongoing. Initially, clinical oncologists will identify prospective participants at The Ohio State University Wexner Medical Center. The clinical oncology team will provide flyers with a brief description of the study to the patients (Multimedia Appendix 1) and will forward patient medical record number information to the research staff upon initial indications of interest.

Subsequently, research staff will verify age and previous diagnosis information via patient chart records in the Epic electronic medical record. Once verified, the contact information will be obtained from Epic patient chart records, and research staff will attempt to contact the participants via a phone call or the messaging system built into MyChart Epic. Research staff will try to contact patients 3 times at precisely 1-week intervals. If participants confirm their willingness to participate, a brief telephone interview will be administered (Multimedia Appendix 2) to screen patients for adherence to the inclusion criteria before obtaining consent (Multimedia Appendix 3) and enrolling participants in the study.

### Devices and Associated Software

#### Consumer-Grade Devices and Software

The Fitbit Charge 6 was chosen for validation because of its widespread popularity, low cost, ease of use, and detailed metrics tracking on a minute-by-minute basis. It is important to note that access to minute-level data collected by Fitbit is not available via a web portal or an app and is only available via an application programming interface (API). There are 2 approaches commonly used to access Fitbit data. The first approach is to use a verified third-party vendor such as Fitabase [27] to manage data collection. The second approach (which will be used in this study) is to create a custom software solution that accesses the Fitbit API (Multimedia Appendix 4). Fitbit uses the OAuth 2.0 authorization framework to grant access to user data [28]. In addition, the Fitbit device requires a mobile phone running iOS (Apple Inc) or Android (Google LLC) for real-time data collection. Participants will be encouraged to use their own devices; however, iPhone 13 (Apple, Inc) devices will be provided to those individuals who do not possess mobile phones.

#### Research-Grade WAMs and Software

For Fitbit validation in the free-living environment, activPAL3 micro and ActiGraph LEAP will be used as the standard research-grade devices for comparison [29]. activPAL3 micro is a thigh-worn monitor with a 14-day battery life and is shown to have strong agreement with the previous generation of ActiGraph GT3X [30]. Proprietary software PALconnect (PAL Technologies) will be used to initialize the devices and download data upon study completion (PALbatch). The ActiGraph LEAP is one of the most comprehensive and accurate research-grade activity monitors currently available, with support for monitoring measures related to PA (eg, step count and minutes of moderate-intensity activity), heart function (eg, heart rate and heart rate variability), and sleep (eg, total sleep time), as well as support for additional measures forthcoming. Proprietary software CentrePoint (ActiGraph LLC) will be used to initialize the devices and download data upon study completion. ActiGraph is easily removable by participants and requires recharging at approximately the 4-day mark when using photoplethysmography features, so both activPAL3 micro and ActiGraph will be used to ensure sufficient data collection for free-living validation. For validation in the laboratory environment, a handheld Sony FX30 digital cinema camera will be used to record participants and compare wearable device outcome measures against gold-standard researcher-labeled video data. Outcome measures from the Fitbit, activPAL3 micro, and ActiGraph devices will all be compared against the researcher-labeled video data. Before participant use, all devices will undergo battery life assessment and functional testing through a 7-day “simulated protocol” conducted by research staff to minimize the risk of device malfunction-related issues, such as incomplete data collection, missing data due to dropped data points, sensor-related impairments leading to unrealistic results (eg, very high activity), excessive noise in the data, etc. This testing protocol will help minimize data quality issues during the actual study period.

### Data Collection and Timeline

#### Overview

**Figure 1:**
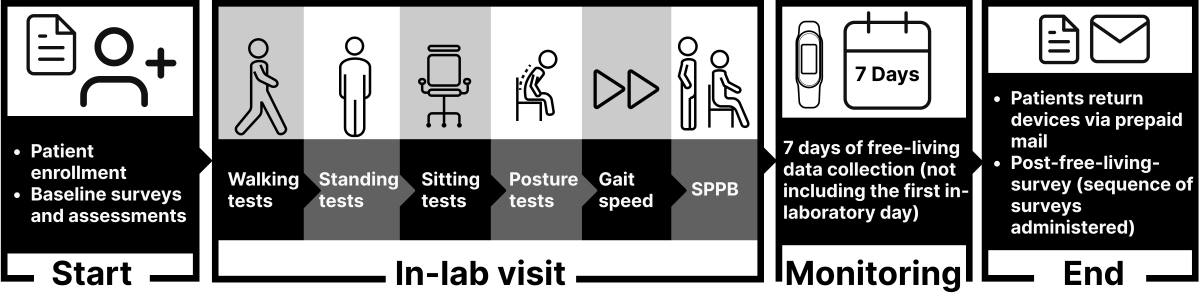
Data collection procedure. SPPB: Short Physical Performance Battery.

The data collection procedure and timeline are shown in Figure 1. Upon enrollment in this study, participants will be emailed a series of surveys to complete before the in-laboratory visit (refer to the Baseline Procedures and Assessments section). Participants will then be scheduled for a 2-hour in-laboratory visit to complete the in-laboratory protocol and receive the activity monitoring devices (refer to the Laboratory Measurement Procedures and Assessments section)*.* Upon completion of the in-laboratory protocol, participants will then be released to their respective free-living environments to further validate the consumer-grade Fitbit device for 7 days, not including the day of the in-laboratory protocol (refer to the Free-Living Measurement Procedures section). Wearable device data collection will take place over 8 days for each participant. Finally, after participants have completed the free-living protocol, they will be sent an email with additional surveys to complete, which will conclude their participation in the study (refer to the Post–Free-Living Survey Procedures section).

#### Baseline Procedures and Assessments

##### Overview

After obtaining patient consent, participants will first be emailed a set of baseline surveys to complete before in-laboratory data collection. Participants will be allowed to complete the surveys before their in-laboratory portion or during the visit. Participants will be permitted to complete the initial set of surveys at home, as allowing participants to complete surveys at home reduces the time required to complete the in-laboratory portion of the protocol. This will be especially beneficial for patients with cancer, who often experience fatigue and might find the lengthy in-laboratory measurement protocol challenging.

##### Instruments and Surveys Administered

Demographic information that will be collected includes full legal name, address, phone number, email, date of birth, ethnicity, race, and sex. Study participants will complete several validated questionnaires to assess quality of life and stress as follows: the Functional Assessment for Cancer Therapy–General 7 (FACT-G7) [31], EQ-5D-5L [32], the Functional Status Assessment [33], and the Perceived Stress Scale [34].

#### Laboratory Measurement Procedures and Assessments

##### Overview

After the baseline surveys are completed, participants will be scheduled for an in-laboratory visit. Before scheduling the visit, a research staff member will screen the participant’s electronic medical record (Epic) to ensure that this visit will be scheduled at least 7 days after the last administration of adjunct therapy; however, oral TKIs and hormonal therapies will be permitted.

##### Previsit Preparation

One day before the laboratory visit, a laboratory-administered Fitbit account will be created for use with the Fitbit devices, and the device will be linked to the custom laboratory-developed Fitbit app to provide real-time API access to the data. The full protocol for API key linkage is available in Multimedia Appendix 4. Although some studies have permitted participants to create their own personal Fitbit accounts, laboratory-administered accounts will be used in this study for data security purposes.

Upon commencement of study data collection, the activPAL3 micro and the ActiGraph firmware will be frozen. Previous studies have indicated that firmware updates can create statistically significant differences in measurements within actigraphy devices [35]. Freezing the firmware reduces variation from firmware-specific differences within the study. Unfortunately, Fitbit does not currently permit firmware freezing.

##### Device Initialization, Application, and Synchronization

After arriving for the in-laboratory protocol, participants’ height and weight will be measured and recorded in REDCap (Research Electronic Data Capture; Vanderbilt University), a secure electronic database. Afterward, research staff will download the custom Fitbit app onto the participant’s mobile device and pair the Fitbit via Bluetooth with the mobile device. During this initialization period, one important but overlooked consideration is ensuring that the participant’s device’s time setting is correctly matched with the computer’s time zone used to initialize ActiGraph and activPAL3 micro, and that automatic updates of time zones are disabled. The free-living protocol requires synchronizing time settings among all 3 devices for accurate validation.

Then, ActiGraph LEAP will be initialized before applying it to the participant using a laboratory computer via CentrePoint software, and the sampling frequency will be set to 32 Hz. Although higher frequencies are available, 32 Hz will be used, as higher sampling rates dramatically reduce battery life, and the 32 Hz frequency has been shown to be sufficient for accurate measurement of PA [36]. With the 32 Hz sampling frequency, we found during testing that the device lasted between 4 and 7 days of continuous wear (using the photoplethysmography sensor significantly reduced battery life). The activPAL3 micro will also be initialized at a sampling rate of 20 Hz to maximize battery life and most closely mirror settings from the ActiGraph.

After initialization, a video recording will be initiated using the Sony FX30 camera. To make it easier for the research team to match the timing of the video recording to the 3 activity monitors, we will record all 3 devices being vigorously shaken by hand, and the exact time (including seconds) of the initialization step will be recorded in REDCap. If the video recording is interrupted (eg, the battery dies and the memory is full) before completion of the in-laboratory protocol, the synchronization step will be repeated.

**Figure 2:**
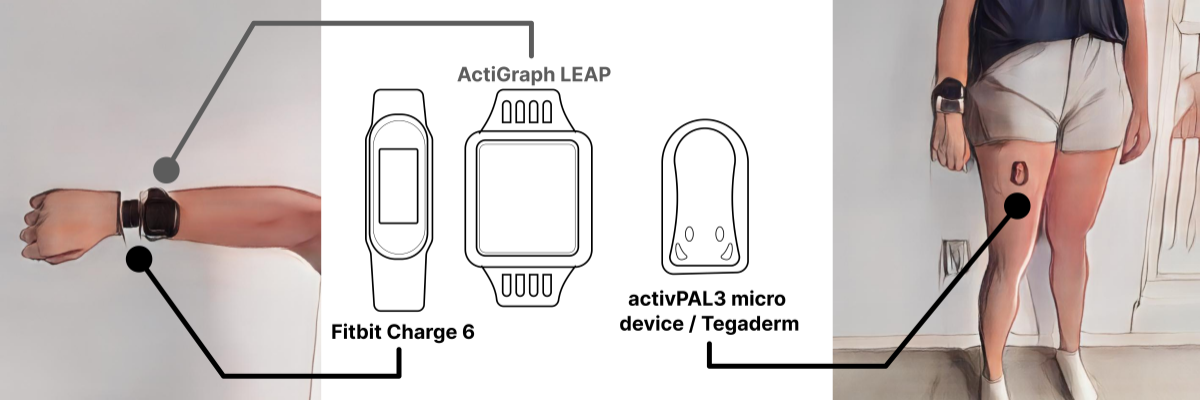
Placement of the Fitbit Charge 6, activPAL3 micro, and ActiGraph LEAP.

After initialization, Fitbit and ActiGraph will be attached to the same nondominant wrist via straps provided by the device’s company. Both devices will be placed on the same wrist to minimize variation from left versus right arm movement during PA. Finally, activPAL3 micro will be attached to the midline anterior aspect of the participant’s right upper thigh using an adhesive waterproof Tegaderm dressing (3M; Figure 2) in concordance with the device instructions and previous studies [37-39].

##### Movement and Posture Change Assessments

**Figure 3:**
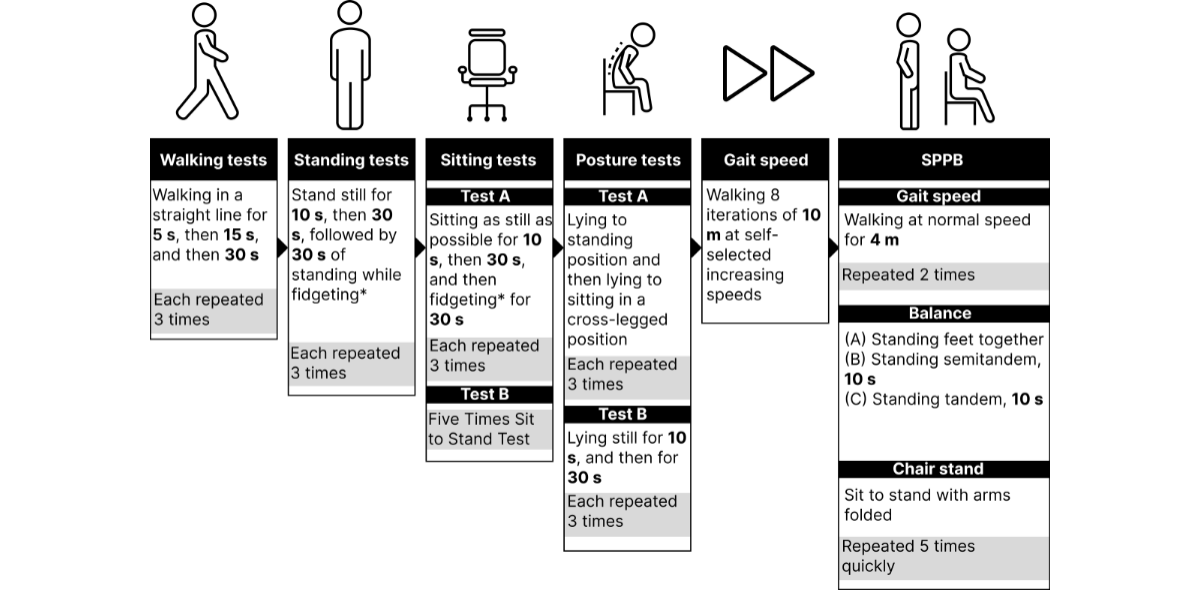
In-laboratory data collection. SPPB: Short Physical Performance Battery.

Following device placement, a battery of tests will be administered (Figure 3) that aligns with validated protocols from previous wearable device studies [20,21].

**Figure 4:**
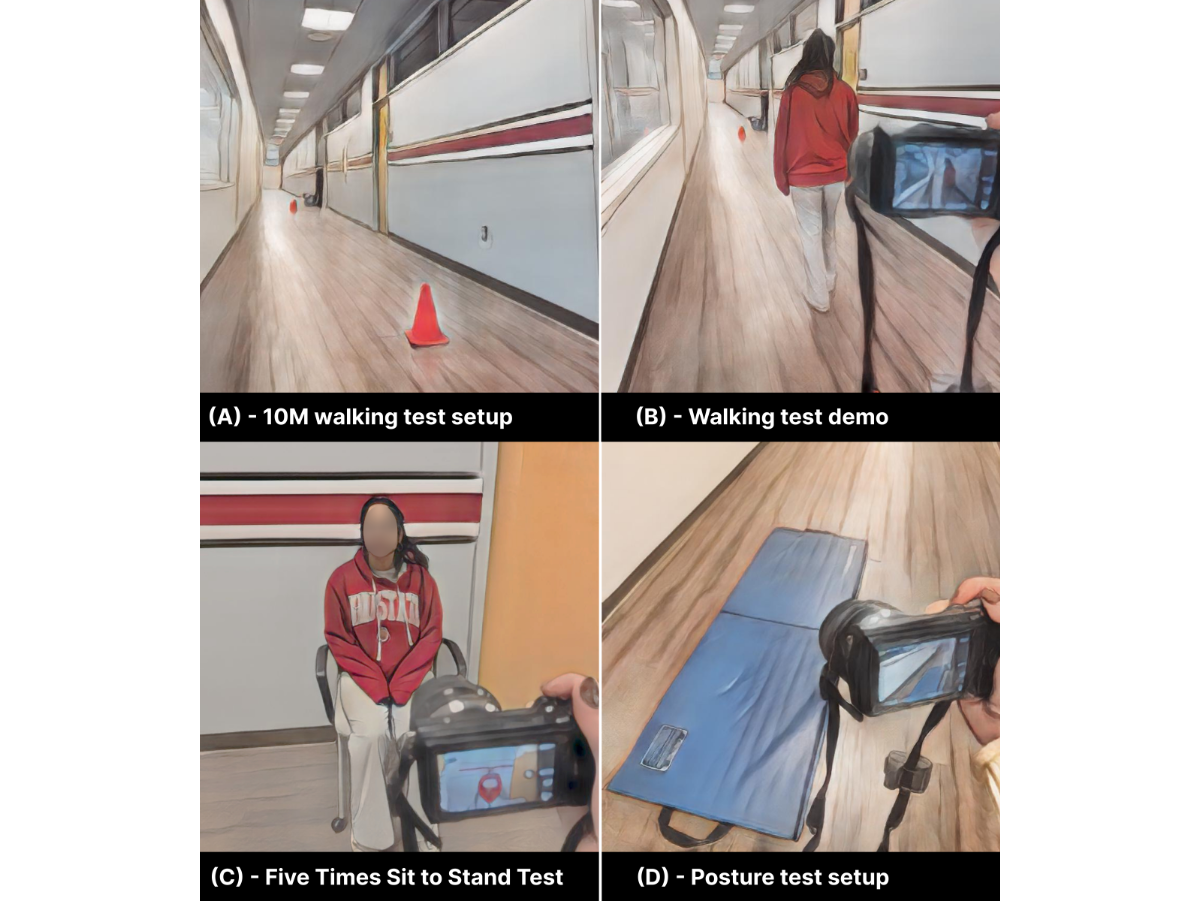
Various data collection setups.

First, a series of variable-time walking tests will be administered. A 10 m walking track will be prepared and marked (Figure 4A). Participants will be instructed to walk for 5 seconds (repeated 3 times), then for 15 seconds (repeated 3 times), and then for 30 seconds (repeated 3 times) for 9 trials (Figure 4B). Step counts from the Fitbit device will be recorded at the beginning and end of each trial. If participants reach the end of the 10 m track, they will be instructed to turn around and continue walking in the opposite direction.

Next, a series of variable-time standing and sitting tests will be administered. First, participants will be instructed to stand as still as possible for 10 seconds (3 trials) and then for 30 seconds (3 trials). Then, the participants will be asked to stand while fidgeting for 30 seconds (3 trials). Following the standing tests, the procedure will be repeated with the participant seated in a chair. The participant will be told to sit as still as possible for 10 seconds (3 trials) and then 30 seconds (3 trials). Then, the participants will be asked to sit while fidgeting for 30 seconds (3 trials).

After the variable-time standing and sitting tests, the five times sit to stand test will be administered. Participants will be instructed to sit in a chair with their backs against the back of the chair (Figure 4C). They will be instructed to cross their arms across their chest, repeatedly stand, and sit for 5 iterations. The time when the participants achieve the standing position on the fifth repetition will be recorded. This test assesses functional lower extremity strength, transitional movements, balance, and fall risk [40].

Following the Five Times Sit to Stand tests, a series of posture change tests will be administered. A thick cushioned mat will be used to alleviate participant burden during this portion of the test to increase participant comfort (Figure 4D). First, participants will be instructed to lie flat on their backs and change their posture to a standing position in any way they can (3 trials). Second, participants will be instructed to lie flat on their backs and change their posture to a cross-legged sitting position in any way they can (3 trials). Third, participants will be instructed to lie as still as possible, flat on their backs, for a 10-second interval (3 trials). Fourth, participants will be instructed to lie as still as possible, flat on their backs, for 30 seconds (3 trials).

Upon the conclusion of the posture change tests, a 10 m gait speed test will be conducted to test the reliability and accuracy of step count measurements at varying walking speeds. Participants will be instructed to walk the length of a 10 m marked walking track at 8 self-selected speeds. Participants will be instructed to walk at the slowest speed during the first trial and gradually speed up in each subsequent trial, with the eighth trial representing the fastest speed. Step counts will be manually recorded from the Fitbit device before and after each of the 8 trials. The final assessment conducted is the Short Physical Performance Battery. The Short Physical Performance Battery is a widely used battery of tests and examinations to assess balance, gait, strength, endurance, and overall lower extremity function [41]. The test examines the ability to stand with feet together side-by-side, in semitandem, and tandem positions; the time to walk 10 m; and the time to rise from a chair and return to the seated position 5 times.

#### Free-Living Measurement Procedures

##### Overview

After the in-laboratory protocol, participants will be instructed to wear the Fitbit Charge 6, ActiGraph LEAP, and activPAL3 micro for 7 continuous days (not including the day of the in-laboratory visit) to validate the Fitbit Charge 6 measurements in a free-living environment.

##### Wear Protocol

In total, 7 days and nights for data collection were chosen, given that previous studies have routinely demonstrated that a 7-day monitoring period provides enough days to achieve a daily intraclass correlation of >80% while capturing both weekday and weekend behavior [42]. Participants will be instructed to always wear the activPAL3 micro and to remove the Fitbit Charge 6 and ActiGraph LEAP when bathing and swimming (but to keep the devices on while showering). Notably, the Fitbit Charge 6 and ActiGraph LEAP do not retain charge for the entire 7-day period. Therefore, patients will be instructed to remove the 2 devices upon depletion of charge and to charge each device for 1 hour. Moreover, the activPAL3 micro does not suffer from such battery-life complications.

##### Monitor Attachment and Participant Instructions

Participants will be instructed on proper wear and application techniques for each device when attached during the in-laboratory protocol. Before leaving the facility, participants will also be given detailed wear instructions (Multimedia Appendix 5). The instruction sheet details the charging and wear information, as well as the contact information of study researchers, to minimize participant burden. The instruction sheet also contains contact information for the research team in the case of adverse events. Furthermore, the activPAL3 micro requires the use of adhesives to stay attached to the participant, and 4 sets of replacement Tegaderm adhesive patches will be provided to each participant. Notably, previous studies have indicated that participants (and particularly those with more sensitive skin, as may be seen in patients with cancer) may experience contact dermatitis from the Tegaderm adhesive used to attach the device [43]. To proactively address potential skin sensitivity issues, kinesiology tape will be provided to participants as a supplemental alternative adhesive option alongside the Tegaderm patches. Providing an alternative method of wearing devices increases wear compliance and reduces participant discomfort during the free-living protocol. Chargers will be provided for both the Fitbit Charge 6 and ActiGraph LEAP.

Finally, in addition to wear instructions, chargers, and replacement Tegaderm and kinesiology tape, participants will also be provided with a sleep and nonwear log to record all periods of sleep and periods when any of the devices are removed (Multimedia Appendix 6). This log will help verify valid periods of wear time during the free-living period.

#### Post–Free Living Protocol Survey Procedures

##### Overview

Upon conclusion of the 7-day free-living protocol, a series of surveys will be emailed to the participant to evaluate the participant’s HRQoL, sleep, symptom burden, and acceptability of the Fitbit Charge 6. These instruments were selected to collect self-reported data corresponding to the period during which participants wore the activity monitor. The collected data will then be used to analyze the relationship among HRQoL, symptom burden, sleep quality, and accelerometry-determined PA. Furthermore, sensitivity analyses will be conducted using these data to assess the impact of these factors on the validity and reliability of the Fitbit device. After completion of the surveys, participants will be instructed to mail the 3 devices back to the laboratory using a provided prepaid envelope. Upon receipt of the devices, a US $60 electronic Amazon gift card will be emailed to the participant as compensation for study participation.

##### Instruments and Surveys Administered

Study participants will complete several validated questionnaires: the FACT-G7 [31], the International Physical Activity Questionnaire–Short Form [44], the Epworth Sleepiness Scale [45], the Pittsburgh Sleep Quality Index [46], the Patient-Reported Outcomes Measurement Information System-29 (version 2.0) [47], and a 31-item technology acceptance survey adapted by Fioranzato et al [48] from Puri et al [49]. These questionnaires were chosen to describe the population and control for potential confounders (eg, symptom burden) that may impact our results.

### Outcome Measures

The primary outcome measures that will be evaluated from the activity monitors are step count; posture and posture changes (activPAL3 micro only); and time spent in light, moderate, and vigorous PA. In the in-laboratory protocol, step count and active time from all 3 devices will be assessed against gold-standard video-recorded manual counting. The posture classification and posture change measurements of activPAL3 micro will similarly be validated against gold-standard video-recorded manual classification (as only the activPAL3 micro records posture and posture changes). In the free-living protocol, measurements of step count and time spent in PA intensities (light, moderate, and vigorous) from the Fitbit will be validated against those recorded by the ActiGraph LEAP and activPAL3 micro.

To account for potential confounding effects, a series of survey-measured outcome measures will also be collected. Before wearable-device data collection, HRQoL will be evaluated across multiple domains. Cancer-related symptoms and concerns, with emphasis on fatigue and ability to enjoy life, will be assessed using the FACT-G7 [31]. General HRQoL will be measured across 5 dimensions (mobility, self-care, usual activities, pain and discomfort, and anxiety and depression) using the EQ-5D-5L [32]. Physical, psychological, social, and role functions will be evaluated using the Functional Status Assessment, a modified version of the Functional Status Questionnaire [33]. Perceived stress levels will be measured using the Perceived Stress Scale, which categorizes stress as low (0-13), moderate (14-26), or high (27-40) based on experiences over the previous month [34]. These measures were included because previous research has shown that HRQoL and mental state can influence movement patterns, potentially affecting activity monitor assessments [50-52].

After the conclusion of the free-living portion of the study, HRQoL will be remeasured using the FACT-G7 survey [31]. PA levels and SB, including time spent sitting, walking, and in various activity intensities, will be assessed using the International Physical Activity Questionnaire–Short Form [44]. Sleep parameters will be evaluated using 2 instruments: the Epworth Sleepiness Scale, which measures general daytime sleepiness, and the Pittsburgh Sleep Quality Index, which generates scores for 7 sleep-related domains (quality, latency, duration, efficiency, disturbances, medication use, and daytime dysfunction) and a global sleep quality score [45,46]. The Patient-Reported Outcomes Measurement Information System-29 will be used to assess overall symptom burden across 8 domains: physical function, anxiety, depression, fatigue, sleep disturbance, social role participation, pain interference, and pain intensity [47]. Wearable tracker acceptance will be evaluated across 6 key dimensions: perceived usefulness, perceived ease of use, privacy concerns, perceived risks, facilitating conditions, and equipment characteristics, using a validated survey adapted for older adult populations [48].

### Data Processing and Quality Control

Immediately upon the receipt of the devices, preliminary data quality control checks will be conducted to ensure that no technical issues resulted in missing or rogue data during the laboratory-based or free living–based data collections as well as proper wear compliance for each device during the field-based data collection. The ActiGraph and activPAL3 micro acceleration data will be checked to determine if the devices are being worn correctly or upside down (as inversion interferes with posture detection), and data will be corrected, when necessary, by inverting them. This is important to note, given that once the data are inverted, they cannot be fed back into the proprietary analysis program and will need to be analyzed using an algorithm developed in-house. Wear compliance will be determined through different approaches across devices. The ActiGraph and activPAL3 micro wear time will be assessed using validated movement-based algorithms [53,54]. Because the Fitbit device does not provide epoch-level activity count data, wear time will be determined using the availability of photoplethysmography heart rate readings, consistent with previous studies [55].

Video data will be considered the gold standard for all laboratory-based validation analysis. For each activity performed by participants in the laboratory, a trained human rater from the research team will manually determine and count each step taken. For movement and posture classification, video data will be organized into 1-second windows for comparison to ActiGraph and activPAL3 micro measures and classified by the human rater as either “active” or “static,” and also as “upright,” “sitting,” “lying down,” or “postural transitions.” Fidgeting while sitting and standing will be categorized as an activity by the rater. The human rater will also manually determine step counts for each activity from the video data.

Similar to the video data classification, accelerometer data from the ActiGraph and activPAL3 micro devices will be organized into 1-second windows. One-minute epochs are the shortest epoch lengths for which the Fitbit data are available to us, and there is no standardized method of human rater classification of activity from video data in 1-minute epochs for activities that last only a small number of seconds. Therefore, the ActiGraph will be used as the criterion measure to evaluate the reliability and validity of the Fitbit device to identify movement and activity intensity levels. Fitbit step counts will be counted by a study team member by manually recording the step counts recorded on the device interface at the start and end of each activity. This will allow us to directly evaluate the agreement of step count measures from all 3 devices to the gold standard video data.

### Statistical Analysis

We will evaluate the validity and reliability of each of the 3 devices following the Food and Drug Administration’s guidance on patient-reported outcome (PRO) measurement instruments as outlined in the study by Byrom et al [56]: (1) content validity, (2) reliability assessment, (3) criterion validity, and (4) ability to detect change.

#### Content Validity

How useful and meaningful these devices are to the patients will be evaluated based on evidence from the peer-reviewed literature, in addition to insights from the wearable tracker acceptance survey administered to patients upon completion of our study.

#### Reliability Assessment

During in-laboratory data collection, intradevice reliability will be assessed by comparing ActiGraph and activPAL3 micro device agreement with the gold standard video data across repeated trials by each participant. For the Fitbit device, intradevice reliability will be assessed by comparing Fitbit agreement with the ActiGraph across repeated trials by each participant. Interdevice reliability will be assessed using the Bland-Altman method and intraclass correlation analyses to compare (1) the total time spent in each posture or movement type as determined by both the accelerometers and video observation [26], (2) the Fitbit and ActiGraph devices’ estimations of movement and different activity intensity levels in 1-minute epochs, and (3) step counts as determined by all 3 devices and video observation. Limits of agreement from the Bland-Altman plots will be used as an additional indicator of reliability in addition to intraclass correlation.

During free-living data collection, interdevice reliability of the Fitbit for measures of active time, step count, and time spent in different activity intensity levels will be compared to the ActiGraph and activPAL3 micro devices using Bland-Altman analyses and intraclass correlation analyses.

#### In-Laboratory Criterion Validity Assessment

The validity of the ActiGraph and activPAL3 micro devices to properly identify different postures and movement will be assessed with agreement (calculated using the Bland-Altman method), sensitivity, specificity, and positive predictive value (PPV) using video data as the gold-standard criterion measure. Sensitivity will describe the percentage of an observation category that is correctly detected by the accelerometers or the ratio of true positives to the sum of true positives and false negatives. Specificity will describe the percentage of observations not belonging to a category that are correctly identified as not belonging or the ratio of true negatives to the sum of true negatives and false positives. PPV will provide the percentage of true positives that is identified when compared to the total number of true positives and false positives determined by the accelerometers. Validity of the Fitbit device to properly identify movement in 1-minute epochs will be assessed with agreement, sensitivity, specificity, and PPV against 2-criterion measures: active time classification in 1-minute epochs from ActiGraph and activPAL3 micro. ActiGraph and activPAL3 micro step counts will be validated against the steps counted manually from the video data for each participant. Agreement, sensitivity, and PPV will be used to assess the research-grade activity monitors’ abilities to accurately detect steps. As mentioned earlier, Fitbit step counts will be evaluated for accuracy against the video data by agreement only, as the validity of each individual step cannot be determined due to the available data granularity.

Moreover, 95% limits of agreement and CIs will be used to determine if there are significant differences in reliability and validity measures among all devices in both the in-laboratory and free-living data collections. In the Bland-Altman plots, systematic error is present if the mean is >0 or <0. Systematic error is considered significant if both repeatability coefficients are on the same side of 0. Linear regression analyses will be used to identify any existing proportional bias.

#### Ability to Detect Change

While a full evaluation of the ability to detect change in outcome measures is out of scope for this study due to the cross-sectional study design, we will calculate the SE of measurement for all intradevice or interdevice assessments. This will inform the ability to detect a minimal detectable change.

### Ethical Considerations

The Ohio State University Cancer Institutional Review Board reviewed and provided initial approval on January 4, 2024 (2023C0190). All participants will provide informed consent before participating in this study. Informed consent materials will be provided in both digital and printed mediums (if requested) and in both written and verbal formats. Informed consent will review, in detail, the study design, components of the study, study duration, right to withdraw from the study, potential risks of participation, protection against risk, and the rights of human research participants. This process will also include the signing of the HIPAA (Health Insurance Portability and Accountability Act) waiver, permitting researchers to review and screen medical records. All patient-identifying information will be kept secure in REDCap. All research data will be anonymized and stored in encrypted Ohio State University Wexner Medical Center–managed hard drives to ensure the highest level of data security and privacy protection. Fitbit API keys will be stored in the laboratory on a single, encrypted machine. For access to Fitbit API data, a custom software solution will be used that only stores API access keys on a local machine. Upon receipt of the devices, a US $60 electronic Amazon gift card will be emailed to the participant as compensation for study participation.

## Results

Data collection is still ongoing. In total, 11 participants have enrolled in the study as of December 2024, with both in-laboratory and free-living data collection completed for 7 (64%) participants. On average, the enrolled participants are aged 63.0 (SD 7.8, range 50.0-73.0) years. Moreover, 8 (73%) participants are women. Data analysis is being conducted simultaneously with ongoing participant enrollment. Participant enrollment is expected to conclude in 2025, and the first reported findings are expected to be disseminated by the end of 2025.

## Discussion

### Summary of Validation Process

This study follows a comprehensive validation protocol designed to assess the accuracy and reliability of both commercial (Fitbit Charge 6) and research-grade activity monitors (activPAL3 micro and ActiGraph LEAP) in LC survivors. The process is divided into two main components: (1) an in-laboratory protocol and (2) a free-living environment protocol.

For the in-laboratory protocol, participants will be fitted with the Fitbit Charge 6, activPAL3 micro, and ActiGraph LEAP and will perform a series of controlled tasks, including walking, sitting, standing, and posture changes. Each task’s outcomes, such as movement, step counts, and postural transitions, as recorded by the research-grade devices, and step counts, as recorded by the Fitbit devices, will be compared to DO (gold standard). This validates the devices’ accuracy in a controlled setting. Following the in-laboratory session, the free-living environment protocol will involve participants wearing the Fitbit Charge 6, activPAL3 micro, and ActiGraph LEAP continuously for 7 days in their daily environments. The Fitbit’s output (eg, activity intensity levels) will be compared to research-grade monitors to assess validity in the laboratory and the free-living environment.

### Challenges in Validation for LC Survivors

Disease- and treatment-related symptoms of LC can influence PA levels in patients. Patients with LC frequently experience debilitating disease-related symptoms, including dyspnea (shortness of breath), cough, fatigue, anxiety, depression, insomnia, muscle wasting, loss of appetite, and pain [57], all of which could impact their ability to engage in PA. To accommodate for the physiologic challenges (eg, reduced pulmonary function) and symptoms inherent to LC, patients might adopt a sedentary lifestyle, which could further contribute to a decrease in cardiorespiratory failure and poorer symptom control, often coined as the “dyspnea-inactivity vicious circle” [58]. These symptoms are exacerbated during and after treatments such as chemotherapy and surgery, further limiting activity levels. Thus, LC survivors spend most of their waking days in sedentary pursuits. When they engage in PA, it is predominantly light-intensity PA (LIPA), with very little time spent in moderate-to-vigorous PA (MVPA), as measured in a previous study using a waist-worn ActiGraph GT3X+ [59]. D’Silva et al [59] objectively assessed SB and PA levels in LC survivors and found that LC survivors spent 9.8 hours per day in sedentary pursuits, 4.1 hours per day in LIPA, and only 14 minutes per day in MVPA. They found that time spent in LIPA was positively associated and sedentary time was significantly and inversely associated with HRQoL and fatigue. However, PA prescriptions for improving symptom burden are based on MVPA [60]. Given the detrimental effects that LC and related treatments have on survivors’ HRQoL, identifying ways to enhance the HRQoL and reduce symptom burden in LC survivors is essential. Using commercial activity monitors is one way that we can start advancing our understanding of how varying PA patterns can influence health outcomes. Thus, there is a need to understand the accuracy and reliability of these devices to capture SB, LIPA, and MVPA levels in LC survivors to advance our understanding of the impact of physical behaviors on PROs.

### Recommendations and Considerations for Validation Studies of Activity Monitors

This section, informed by findings from the ongoing study, summarizes recommendations and considerations for validation studies of activity monitors (Textbox 1).

**Recommendations and considerations.**

**Validation protocol recommendations**

- Use research-grade (activPAL3 micro and ActiGraph LEAP) and commercial (Fitbit Charge 6) monitors for cross validation in the laboratory and free-living environments.
- Use multiple research-grade devices (activPAL3 micro and ActiGraph LEAP) if feasible to ensure sufficient data collection during free-living validation.
- Direct observation in the laboratory should serve as the gold standard for validating physical activity (PA) measurements.
- Surveys should be administered to evaluate potential psychosocial and physiological confounders impacting PA behaviors.
- Provide participants with the ability to complete survey instruments at home to reduce the burden during in-laboratory visits.

**Data management and software recommendations**

- Use third-party tools or custom software to manage data.
- Store application programming interface keys on local, secure machines to minimize the risk of data breaches, or use a secure third-party Fitbit data access vendor such as Fitabase.
- Create laboratory-administered accounts when using consumer-grade activity monitors to minimize the risk of data breaches.
- Freeze firmware on research-grade devices to prevent inconsistencies from software updates.
- Adjust sampling rates for devices based on the length of the free-living observation period.
- Test all devices and software before launching the study.

**Participant enrollment recommendations**

- Recruit 30% more participants to account for noncompliance and data collection errors.
- Partner with medical professionals to understand disease- and treatment-related factors that could impact the behavior being evaluated (eg, PA) and to support recruitment efforts.

**Device handling and wear recommendations**

- Synchronize devices’ time settings for accurate data matching and log this using a secure electronic database (eg, REDCap [Research Electronic Data Capture; Vanderbilt University]).
- Minimize variation caused by body placement by placing all monitors in identical locations (eg, dominant vs nondominant wrist) when feasible.
- Provide participants with sleep and nonwear logs to validate sleep-based metrics and periods of nonwear.
- Evaluate additional environmental factors impacting the device’s performance (eg, temperatures).
- Provide a prestamped envelope to reduce participant burden once the data collection is complete, and provide additional tape to seal the envelope to avoid loss of devices in transit.
- Permit participants to wear water-resistant devices while showering to reduce the burden.

**Participant comfort recommendations**

- Demonstrate how to properly use (eg, wear and charge) the wearable and provide instructions to reduce any negative experiences (eg, confusion and anxiety).
- When applicable, provide alternative adhesives (eg, kinesiology tape instead of Tegaderm) for participants to increase comfort and improve device wear compliance.

**Statistical recommendations**

- Perform preliminary checks on data for compliance and wear accuracy during (if possible) and immediately after receiving the devices.
- Consider extending the data collection period from 7 days to 10 days. This would provide more variability in daily activity patterns, especially capturing potential differences between weekdays and weekends.
- After data collection, follow up with participants to debrief about their experience wearing the devices, focusing on any difficulties encountered.

When designing validation protocols for activity monitors, a combination of research-grade devices such as the activPAL3 micro and ActiGraph LEAP, along with commercially available devices such as the Fitbit Charge 6, should be used to ensure robust cross validation in both controlled laboratory settings and free-living environments. DO is often considered the gold standard for assessing the accuracy of WAMs due to its accuracy and objectivity and should be implemented whenever possible [20]. It allows researchers to record PA behaviors, such as steps, distance, or activity duration, with high fidelity and without relying on potentially flawed device algorithms or self-reported data. Unlike wearables, DO is independent of biases caused by sensor misalignment, body size, skin tone, or activity type. It also provides rich contextual information about activities, such as intensity, posture, and transitions, which devices may not fully or accurately capture. However, DO has limitations, including being labor intensive, requiring considerable time and resources, and being restricted to controlled settings or small sample sizes. There is also potential for observer bias if strict protocols are not followed. Because of these challenges, many validation studies (such as ours) combine DO with other methods, such as accelerometer-based measures, video analysis, or doubly labeled water, to comprehensively evaluate wearable performance. In addition, to improve the data quality, the activities chosen for the in-laboratory protocol must represent those that occur in the real-world environment for the population being studied, as accuracy is influenced by shorter task segments and slower and higher gait velocities. Moreover, some validation studies use treadmills during the in-laboratory procedures, which may impede individuals from walking naturally and at their normal walking speed. As stated in the Free-Living Measurement Procedures section, many psychosocial and physiological factors may confound PA levels and the ability of the device to capture information accurately, such as mental and physical PROs (eg, fatigue and pain). Thus, researchers should administer surveys to participants to assess potential. We recommend allowing participants to complete these before or after the appointment to reduce the burden during the in-laboratory visit.

From a data management perspective, using third-party tools or custom software to handle device data is advisable, ensuring that API keys are stored securely on local machines to minimize the risk of data breaches. At the same time, researchers should aim to maintain consistency by freezing the firmware on research-grade devices to avoid measurement variability caused by software updates. To promote interoperability, common standards for storing mobile health data, such as Fast Healthcare Interoperability Resources, Health Level 7, and open mobile health schemas, should be used to enable consistent data exchange and integration across platforms and systems [61]. Finally, one of the most important practices is to test all devices thoroughly before the study commences.

Regarding participant recruitment, we recommend planning for the possibility of having to enroll 30% more participants than initially required to account for potential noncompliance and data collection errors. Collaborating with medical professionals will (1) enhance recruitment and (2) provide insights into disease- and treatment-related factors that may influence PA behaviors, ultimately impacting the device’s ability to capture physical behaviors. Recruitment could be enhanced by leveraging the patient–health care provider relationship by having the health care provider present the study to the patient during their encounters. In addition, the health care provider can help prescreen participants given their understanding of participants’ eligibility related to personal (eg, age, sex, and willingness to participate in the research study) and clinical factors (eg, ability to walk independently and disease status), which can reduce the number of eligibility screen fails and burden on the research team.

Proper device handling is crucial; synchronizing time settings across devices and documenting this information in a secure electronic database (such as REDCap) ensures accurate data matching. Multiple research-grade monitors should be considered for free-living conditions to guarantee continuous data collection, especially given battery limitations. Batteries can be limited by the participant’s baseline activity levels or the use of an activity monitor (eg, tracking activities and scrolling through its features). Cold temperatures can also diminish battery function, so it is essential to consider this depending on the geography and season where these monitors are being used. Participants should be provided with sleep and nonwear logs to help validate metrics related to sleep and periods of nonwear, and additional factors, such as environmental conditions, should also be evaluated for their impact on device performance. For water-resistant devices, we recommend allowing participants to shower while wearing the devices, as this simplifies wear instructions and reduces participant burden.

Finally, participant comfort must be prioritized to improve compliance. For example, to improve the chances that the participant adheres to the wear protocol, we have found that it is important to provide participants with clear demonstrations on how to wear and charge the devices and provide alternative adhesives (eg, kinesiology tape in addition to Tegaderm) to minimize discomfort and align with their preferences (ie, Tegaderm can create a waterproof barrier that allows users to wear the devices during activities involving water). Statistically, researchers should perform preliminary checks on the data for compliance and wear accuracy, both during the study and immediately after receiving the devices. Extending the data collection period from 7 to 10 days can also capture more variability in daily activity patterns, especially distinguishing differences between weekdays and weekends. Pre–data collection reminders about the appointment as well as reminding them of key activities (eg, returning the device and survey completion) can improve participant compliance. Post–data collection follow-up with participants could also be beneficial, allowing an additional opportunity to gather feedback on their experience with the devices, address any issues encountered during the study, and review the monitor wear log.

### Integration and Impact of Commercial Activity Monitors in Health Care and Patient Management

The integration of commercial activity monitors into health care holds significant promise, particularly in enhancing patient outcomes and supporting the long-term management of chronic conditions such as cancer. Commercial wearables such as Fitbit and other accelerometer-based devices have demonstrated their potential in monitoring PA, circadian rhythm, sleep, and other vital health metrics, providing real-time patient and clinician data. In cancer care, including clinical trials, wearables are increasingly being used to monitor and support the delivery of PA interventions aimed at improving health outcomes (eg, HRQoL) during and after treatment, as shown by studies across cancer sites (ie, breast, gastrointestinal, lung, and gynecologic) [62]. These devices provide increased accessibility, ease of use, and the ability to gather health data outside clinical settings in near real time.

One notable advantage of commercial wearables is their ability to seamlessly integrate into patients’ daily lives, offering a nonintrusive and ecologically valid approach to monitoring activity and other health metrics. These data can be used to tailor interventions, improve patient adherence to exercise regimes, enhance symptom tracking, and detect adverse events related to the disease and treatment [62,63]. Electronic health record (EHR) systems such as Epic already incorporate wearable data through API integrations, allowing clinicians to view patient-generated digital health data. This capability enhances the continuity of care by providing a comprehensive view of patient health, particularly between clinical visits [5]. In addition, insurance companies such as United Healthcare and Humana incentivize the use of wearables by offering rewards and health-tracking programs that integrate wearable data into personal health records. These initiatives promote proactive health management, encouraging patients to engage with their health data and use them to inform daily health behaviors [5]. In addition, the potential to link wearable data with PROs and leverage machine learning approaches enables a more holistic view of a patient’s health, offering predictive insights that extend beyond PA patterns to include mental health and HRQoL [62,63].

However, several considerations must be made when using wearables in health care. One challenge is adherence, as adherence rates vary widely across studies, with patients sometimes wearing devices inconsistently [62,64,65]. This inconsistency can skew data and make it difficult to draw accurate conclusions about a patient’s health over time. Therefore, it is important to standardize what is considered a valid day for PA measures, often denoted as at least 10 hours of wear per day [64]. The variability in measurement accuracy across different devices presents a notable limitation, particularly for complex metrics such as MVPA. The reliability of MVPA data is crucial, as it is the foundation of the Physical Activity Guidelines for Americans and is directly linked to health outcomes [66]. Depending on the device (brand, model, and firmware) and population, studies have shown variable accuracy in tracking activity in free-living conditions between commercial monitors and research-grade devices in PA metrics such as step counts and moderate-to-vigorous activity (often overestimated in those with mobility issues) [64,65,67,68]. Therefore, while commercial wearables offer a promising tool for monitoring patient health, standardized data collection and interpretation protocols are needed to ensure their reliability in clinical settings.

The future of commercial wearables in health care largely depends on their seamless integration into broader health systems, mainly by incorporating wearable data into EHRs. As EHRs evolve, the ability to merge real-time data from wearables with clinical records could provide clinicians with continuous, detailed insights into a patient’s health status, enabling more personalized care and timely interventions (eg, to support unmet needs) [5,69]. This integration is especially promising in oncology, where wearables are explored in clinical trials to monitor treatment-related outcomes and improve patient management. Studies have shown a growing interest in using wearables to track PA, circadian rhythms, and other health metrics in patients with cancer undergoing treatment [62,63]. As technology continues to evolve, commercial wearables have the potential to significantly contribute to the move toward precision medicine by collecting real-world, continuous data that can be used to tailor clinical care and interventions.

### Study Protocol Strengths and Limitations

This study presents several strengths. Building on previous research [20,21], this protocol addresses inconsistencies in metrics, wear time, and definitions of valid data by providing extensive supplementary materials, including API code, wear instructions, nonwear logs, and consent forms, to promote standardization and reproducibility. A key feature of the study is its combination of laboratory-based validation, using DO as the gold standard, with assessments in real-world, free-living conditions. This dual approach enhances the reliability and applicability of the findings. In addition, the study focuses on LC survivors, a high-prevalence but underresearched population, recognizing their unique challenges. Rigorous methods, such as freezing firmware and software updates, are used to minimize measurement variability, and this protocol includes tracking nonwear periods to ensure comprehensive data collection and insights into wear compliance. Despite its strengths, this study also has limitations. It does not evaluate device accuracy during active cancer treatments, such as chemotherapy or radiation therapy, with the exception of specific cases, such as TKIs, which may miss crucial activity changes during these periods. While the study duration is standard, it is relatively short, limiting the assessment of device reliability and ability to detect change as well as participant compliance over longer periods in real-world conditions. Furthermore, the findings may be influenced by environmental and contextual factors, such as temperature, humidity, and daily routines, which were not thoroughly examined.

### Conclusions

This paper provides a comprehensive protocol for validating consumer-grade and research-grade WAMs in LC survivors, addressing a critical gap in the literature. By combining rigorous laboratory-based assessments with real-world, free-living evaluations, this protocol highlights the strengths and limitations of these devices in capturing PA and SBs in a uniquely vulnerable population. The inclusion of LC survivors underscores the importance of tailoring validation methods to the specific needs and challenges of underrepresented groups in health research. While this study offers valuable insights into wearable device performance, further research is needed to explore device reliability over extended periods, during active cancer treatments, and under diverse environmental conditions. These findings provide a foundation for future studies aiming to standardize validation protocols and advance the integration of wearable technologies in oncology care, ultimately supporting improved health outcomes for cancer survivors.

## Appendix

Multimedia Appendix 1 Study material used.

Multimedia Appendix 2 Telephone interview and script.

Multimedia Appendix 3 Consent documentation.

Multimedia Appendix 4 Fitbit application workflow.

Multimedia Appendix 5 Activity monitor wear instructions.

Multimedia Appendix 6 Sleep and nonwear diary.

Multimedia Appendix 7 SPIRIT (Standard Protocol Items: Recommendations for Interventional Trials) checklist.

## Abbreviations

API: application programming interface
DO: direct observation
EHR: electronic health record
FACT-G7: Functional Assessment for Cancer Therapy–General 7
HIPAA: Health Insurance Portability and Accountability Act
HRQoL: health-related quality of life
LC: lung cancer
LIPA: light-intensity physical activity
MVPA: moderate-to-vigorous physical activity
PA: physical activity
PPV: positive predictive value
PRO: patient-reported outcome
REDCap: Research Electronic Data Capture
SB: sedentary behavior
TKI: tyrosine kinase inhibitor
WAM: wearable activity monitor

## Data Availability

The datasets generated or analyzed during this study are available from the corresponding author on reasonable request. The SPIRIT (Standard Protocol Items: Recommendations for Interventional Trials) checklist based on the SPIRIT guidelines is available in Multimedia Appendix 7.
