# Supplementary figures and images for: Comparing the Accuracy of Different Wearable Activity Monitors in Patients With Lung Cancer and Providing Initial Recommendations: Protocol for a Pilot Validation Study
Source: JMIR Res Protoc. 2025 Jun 19;14:e70472. doi: 10.2196/70472 (PMC12226780; doi:10.2196/70472)

## Supplemental File 1: Recruitment Flyer

**
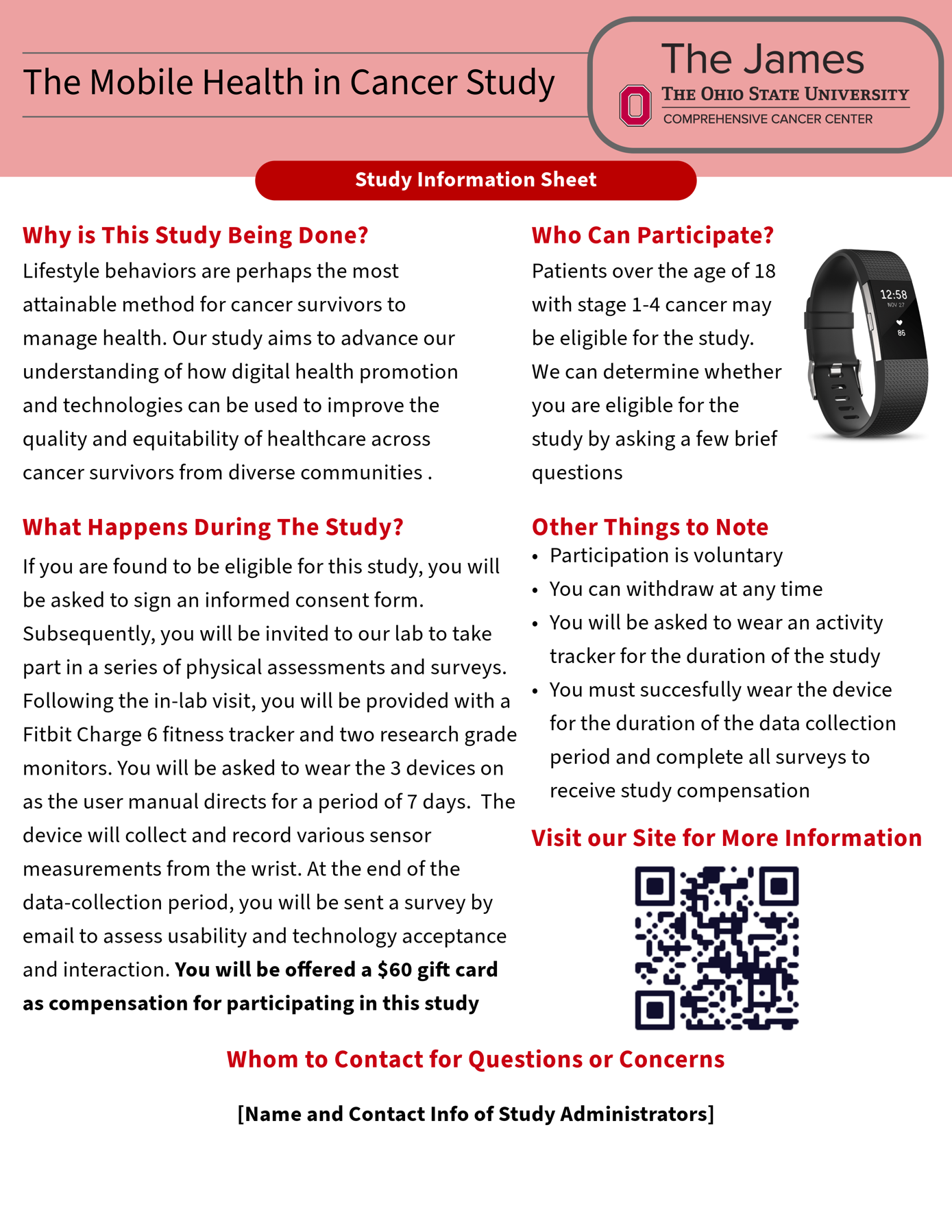
**

Supplement: Multimedia Appendix 1 [file resprot_v14i1e70472_app1.docx]
