# Supplementary material for: Comparing the Accuracy of Different Wearable Activity Monitors in Patients With Lung Cancer and Providing Initial Recommendations: Protocol for a Pilot Validation Study
Source: JMIR Res Protoc. 2025 Jun 19;14:e70472. doi: 10.2196/70472 (PMC12226780; doi:10.2196/70472)
Supplement: Multimedia Appendix 2 [file resprot_v14i1e70472_app2.docx]

*[Interviewer Script]: Hello, my name is ________. I am from The Ohio State University Comprehensive Cancer Center. I’m trying to contact [patient name], is this Mr/Ms. [patient name]?*

1. If “NO”: *May I speak with [insert patient name]?*

- if patient is not available: *Please have them call [insert your name], at [your number]. Thank you and have a great day.* *End call*

2. If “YES” and talking to the patient:

- *I am contacting you because you indicated to [Provider Name] that you may be interested in participating in our research study investigating the impact of fitness, lifestyle, and activity on outcomes for cancer patients and survivors. I am calling from The Ohio State University Comprehensive Cancer Center to see if you would be interested in learning more about and potentially participating in this research study?*
- If “NO”: *Thanks for the phone call and have a great day!* *End call*
- If “YES”: *Great, is this a good time to talk?*
- If “NO”: *Please let me know when the best time to call again would be.* *End call*

3. If it is a good time to talk: *Our study is interested in promoting healthy lifestyle recommendations, like regular physical activity and healthy eating patterns, for cancer survivors like you. Evidence shows that adhering to the lifestyle recommendations posed by institutions such as the American Cancer Society and the American Institute of Cancer Research can lead to reduced symptom burden, improved health outcomes, and health-related quality of life among adults. These guidelines are centered on three main recommendations which include:*

1. *Engage in regular physical activity*
2. *Consume a healthy dietary pattern*
3. *Avoid obesity*

*However, we recognize that there are many factors related to survivorship that can make it difficult for cancer survivors to adopt healthy lifestyle habits. The goal of our study is to investigate the association between the usage and interest in mHealth/fitness technology and how it can potentially improve health behaviors. One of the main components of this study and what you’ll be participating in is an investigation to assess and compare the results of lifestyle data obtained from a commercial fitness tracker*

*The study will have three components; a 2-hour visit to our lab where you will complete a series of surveys, and physical assessments, a 7-day free-living period where we will give you a set of three activity monitors to wear while you go about your daily life, and a final post-observation survey which will be completed virtually from your home and takes 20 minutes to complete.*

*Participation is voluntary and participants that enroll in our study may back out at any time. You will be compensated with a $60 gift card upon completion of the study.*

*If you are interested in participating in our study, the next step will be to complete an initial set of screening questions to determine your eligibility for our study. I just have to ask you a few screening questions, where we will collect preliminary data to ensure your eligibility. Your responses to these questions will be kept completely private and will not be shared with your doctor, other healthcare providers, or anyone except for research personnel. Do you have any questions about the study?*

4. If “YES”: **Provide appropriate responses to their questions**

- If “NO”, *C*ontinue to number 5 if screening has not yet been completed. Otherwise continue to number 6 if screening was previously completed**

5. *Would you like to see if you qualify for the study?*

- If “NO”: *Thanks for the phone call and have a great day!* *End call*
- If “YES”:
- *I will need to confirm your identity. Please provide your month and year of your birthdate.*
- *I would also like to ask you some detailed questions to see if you are eligible to participate. This will take just a few minutes of your time. Do I have permission to continue?*
- If “YES”: *continue with the Aim2B - Screening Survey*

6. *Thank you for answering these questions.*

__ Not Eligible (If any ineligible boxes are checked):  *From the answers provided, it appears that you are not eligible to participate in this study. However, I greatly appreciate you taking the time to speak with me today. As I stated before, the information you provided today will be kept confidential. Do you have any questions for me?*

- If “NO”: *Thank you for your time and have a great day!* *End call*
- If “YES”: *Answer any questions or concerns from survivor. Then* *Thank you for your time and have a great day!* *End call*

__ Eligible (continue with phone call): *Great! It looks like you are eligible to participate in our study. I can provide you with some more details if you would like and we can go ahead and schedule your first visit. Would you like me to continue?*

- If “YES”: *The study will be conducted in three phases. A timeline for each phase will be provided below:*
- *Lab visit: You will be asked to come to the lab for a 2-hour visit. During that visit, you will be given a series of surveys and physical assessment tests that involve sitting, standing, and walking*
- *Free-living: After the lab visit, you’ll be given a set of three activity monitors to wear. You’ll be asked to wear these monitors on your wrist for a period of 7 days as you go about your daily lives*
- *Post-observation surveys: Finally, after the 7-day period from above, you will be asked to complete a set of survey questions virtually. You will also be given a prepaid envelope to mail the fitness trackers back. Once you complete the study, we will send you a $60 gift card as compensation for study participation*

*Do you have any other questions that I can answer?* **Answer questions**

7. *Would you like to participate in this study?*

- If “NO”: *Thanks for taking the phone call and have a great day!* *End call*
- If “YES”: *We can either schedule an appointment to go over the study and complete the consent form virtually or over the phone at a later time, or we can go through the process now. What would you like?*
- If “APPOINTMENT”: **Schedule appointment, enter into REDCap**
- If “NOW”: **Proceed to the Aim2B – Consent and Scheduling Recruitment Script. Enter current date into REDCap**

8. *Thank you for taking the phone call and have a great day!* *End call*
