# Supplementary material for: Comparing the Accuracy of Different Wearable Activity Monitors in Patients With Lung Cancer and Providing Initial Recommendations: Protocol for a Pilot Validation Study
Source: JMIR Res Protoc. 2025 Jun 19;14:e70472. doi: 10.2196/70472 (PMC12226780; doi:10.2196/70472)
Supplement: Multimedia Appendix 3 [file resprot_v14i1e70472_app3.docx]

**The Ohio State University Combined Consent to Participate in Research and HIPAA Research Authorization**

| **Study Title:** |  |
| --- | --- |
| **Principal Investigator:** |  |
| **Sponsor:** |  |

- **This is a consent form for research participation.** It contains important information about this study and what to expect if you decide to participate. Please consider the information carefully. Feel free to discuss the study with your friends and family and to ask questions before making your decision whether or not to participate.
- **Your participation is voluntary.** You may refuse to participate in this study. If you decide to take part in the study, you may leave the study at any time. No matter what decision you make, there will be no penalty to you and you will not lose any of your usual benefits. Your decision will not affect your future relationship with The Ohio State University. If you are a student or employee at Ohio State, your decision will not affect your grades or employment status.
- **You may or may not benefit as a result of participating in this study.** Also, as explained below, your participation may result in unintended or harmful effects for you that may be minor or may be serious depending on the nature of the research.
- **You will be provided with any new information that develops during the study that may affect your decision whether or not to continue to participate.** If you decide to participate, you will be asked to sign this form and will receive a copy of the form. You are being asked to consider participating in this study for the reasons explained below.

**Key Information About This Study**

- You will be participating in a study that is aiming to test how accurate and precise certain devices are compared to one another. These will include activity monitors, both research-grade, high-tech devices along with regular consumer products. The goal is to evaluate the differences between the high-tech research-grade devices and the regular consumer products that you could buy at a store. The devices we will be using are listed below:
  - Fitbit: A consumer grade wrist-worn activity monitor
  - activPAL: A research grade thigh-worn activity monitor
  - ActiGraph: A research grade wrist-worn activity monitor
- During the study you will be asked to:
  - *Baseline Procedures:*
    - Complete a series of 4 surveys
    - Wear the different devices while participating in different physical activities (standing, walking, sitting, etc)
  - *7-day Trial Period:*
    - You will be given 3 activity monitors to wear at home while you go through your everyday lives
      - Activity Monitors:
        - Actigraph LEAP
        - ActivPAL
        - Fitbit
    - You will be asked to mail back the devices at the end of the 7 day period, using the provided envelope and stamps
  - *Post-7-day Period:*
    - You will be sent another series of 6 surveys to be completed at home at the end of the 7 day period

**1. Why is this study being done?**

There is strong evidence to support the positive effect of healthy living on reducing symptoms, improving quality of life, and reducing the risk of developing cancer. However, survivors oftentimes find it difficult to follow guidelines for a variety of reasons. One way to potentially overcome this challenge is to use consumer technology (like a Fitbit) to give survivors the ability to track and engage in healthy behaviors. Our study aims to better understand the level of accuracy in consumer devices like Fitbit, compared to research-grade devices like Actigraph, and activPAL. By understanding the differences in these devices, we can better understand how to potentially use these devices to improve lifestyle behaviors and quality of life.

**2. How many people will take part in this study?**

We will enroll 15 lung cancer survivors, 15 prostate cancer survivors, and 15 colorectal cancer survivors into the study.

**3. What will happen if I take part in this study?**

There will be several parts to this study:

- First, you will be asked to complete a series of surveys and questionnaires. These questions will focus on your physical activity levels, cancer-related symptoms, and stress levels. The questions can be completed online via a survey link sent to your email address prior to your visit, or during your study visit itself.
- Second, you will be invited to come to our study site at the Ohio State University for a 2-hour visit. You will then be asked to wear three separate activity monitors (the activPAL, the Fitbit, and the ActiGraph). While wearing the monitors, you will be asked to complete a series of basic physical tasks including walking at various speeds, sitting, and standing. As you carry out these tasks, we will take a video and audio recording of you completing the different physical activities. This will allow the research team to compare the measurements obtained by the activity monitors to the actual movements that took place as seen on video.
- After the study-site component is complete, you will be sent home while still wearing the three activity monitors. You will be asked to go about your daily life for 7 days while wearing the 3 activity monitors. You will be asked to wear the monitors for 24 hours a day during this period. You will also be given an instruction sheet with directions on how to care for the devices. If you do not have a smartphone, we will also provide you with an iPhone to use for the duration of the study. This iPhone will have restricted access, but will enable you to use the Fitbit app and make phone calls/text messages in case you need to reach the research team. After the conclusion of the 7-day observation period, you will be asked to mail the smartphone back to the OSU study team using a prepaid envelope that we will provide
- After the 7-day period is over, you will then be asked to complete a final set of surveys. These surveys will be delivered online to you via a secure email link, and will take approximately 20 minutes to complete. After completing the surveys, you will then be provided with a prepaid envelope to send the 3 activity monitors [and the iPhone, if you were provided with one] back to our lab. Once we receive the 3 activity monitors and you have completed the surveys, we will mail you a $60 Amazon gift card.

**4. How long will I be in the study?**

You will be in the study for 8 days. During the first day, you will be asked to come into our study site for 2 hours and to complete a set of surveys (either prior to coming into the lab or at our lab itself). For days 2 through 8, you will wear the monitors and continue with your everyday activities. Finally, you will virtually complete a final set of surveys on day 8, which will take 20 minutes to complete.

**5. Can I stop being in the study?**

You may leave the study at any time. If you decide to stop participating in the study, there will be no penalty to you, and you will not lose any benefits to which you are otherwise entitled. Your decision will not affect your future relationship with The Ohio State University.

**6. What risks, side effects or discomforts can I expect from being in the study?**

This is an observational study with minimal associated risks. Some possible risks include:

- Possible rare cases of contact dermatitis from the activity monitor or adhesive (e.g., Tegaderm or Kinesiology Tape).

There are minimal physical risks for taking part in this study. We will not be asking you to perform any physical activities or exercise during the at-home portion of the study; you will only be asked to wear the tracker and continue with your regular activities.

If you experience any psychological or other discomfort, we will address such emotional reactions and refer you, as needed, to appropriate services. If you experience extreme acute or persisting affective reactions during the study period, you will be referred to a psychiatrist or clinical psychologist associated with the site. If any of the questions on the surveys you will take cause psychological discomfort, you have the option to not take the survey or to stop taking the survey at any time.

An additional potential risk is the event of a breach of confidentiality. To minimize the likelihood of this, all research staff will be properly trained, and data will be stored on password-protected Ohio State Wexner Medical Center secure encrypted computers, on secure hard drives. Personal health data will be stored behind the Ohio State Wexner Medical Center firewall on Ohio State Wexner Medical Center secure encrypted computers. Along with protection measures, only limited research staff will have direct access to the restricted data. We will work to make sure that no one sees your online responses without approval. But, because we are using the Internet, there is a chance that someone could access your online responses without permission. In some cases, this information could be used to identify you.

**7. What benefits can I expect from being in the study?**

There will be no direct benefit. It is possible, however, that you may become more aware of your lifestyle behaviors and perhaps take action to improve them. Also by advancing our understanding of the ease of use of fitness trackers in cancer care, along with the difference in research-grade technology, we expect that the findings from this study will inform related research (digital health technology and interventional use) in cancer care.

**8. What other choices do I have if I do not take part in the study?**

You may choose not to participate without penalty or loss of benefits to which you are otherwise entitled.

**9. What are the costs of taking part in this study?**

There are no costs to you to participate in this study. You will be asked to mail the devices back to the lab at the end of the trial; however, the research staff will provide the envelopes and stamps required.

**10. Will I be paid for taking part in this study?**

By law, payments to participants are considered taxable income.

Upon completing all surveys, completing the in-lab visit, and returning all 3 activity monitors, we will provide you with a $60 Amazon gift card.

**11. What happens if I am injured because I took part in this study?**

If you suffer an injury from participating in this study, you should notify the researcher or study doctor immediately, who will determine if you should obtain medical treatment at The Ohio State University Wexner Medical Center.

The cost for this treatment will be billed to you or your medical or hospital insurance. The Ohio State University has no funds set aside for the payment of health care expenses for this study.

**12. What are my rights if I take part in this study?**

You will be provided with any new information that develops during the course of the research that may affect your decision whether or not to continue participation in the study.

You may refuse to participate in this study without penalty or loss of benefits to which you are otherwise entitled. If you are a student or employee at Ohio State, your decision will not affect your grades or employment status.

If you choose to participate in the study, you may stop participation at any time without penalty or loss of benefits. You can also decline to answer any questions in the survey that you wish not to answer. By agreeing to participate, you do not give up any personal legal rights you may have as a participant in this study.

An Institutional Review Board responsible for human subjects research at The Ohio State University reviewed this research project and found it to be acceptable, according to applicable state and federal regulations and University policies designed to protect the rights and welfare of research participants.

**13. Will my de-identified information be used or shared for future research?**

Yes, it may be used or shared with other researchers without your additional informed consent.

**14. Will my study-related information be kept confidential?**

Efforts will be made to keep your study-related information confidential.

We will work to make sure that no one accesses your responses without approval. We will remove any identifying information and replace it with a study ID number. Your information will not be shared with anyone outside our research study team.

Paper-based versions of consent forms and surveys will be stored in a locked drawer or cabinet in an investigator’s locked office. Any electronic audio or video files will be maintained on a password-protected secure encrypted computer maintained in a designated drive behind the OSU Wexner Medical Center firewall. Any physical activity data collected through Fitbit, ActivPAL, and Actigraph will be stored behind the Ohio State Wexner Medical Center firewall.

However, there may be circumstances where this information must be released. For example, personal information regarding your participation in this study may be disclosed if required by state law. Also, your records may be reviewed by the following groups (as applicable to the research):

- Office for Human Research Protections or other federal, state, or international regulatory agencies;
- The Ohio State University Institutional Review Board or Office of Responsible Research Practices;
- The sponsor, if any, or agency (including the Food and Drug Administration for FDA-regulated research) supporting the study.

**15. HIPAA AUTHORIZATION TO USE AND DISCLOSE INFORMATION FOR RESEARCH PURPOSES**

1. **What information may be used and given to others?**

- Past and present medical records;
- Research records;
- Records about phone calls made as part of this research;
- Records about your study visits (including video-recordings taken during the in-lab procedures);
- Information that includes personal identifiers, such as your name, or a number associated with you as an individual
- Health data accessed from medical records for research purposes

1. **Who may use and give out information about you?**

Researchers and study staff.

1. **Who might get this information?**

- The sponsor of this research. “Sponsor” means any persons or companies that are:
  - working for or with the sponsor; or
  - owned by the sponsor.
- Authorized Ohio State University staff not involved in the study may be aware that you are participating in a research study and have access to your information;
- If this study is related to your medical care, your study-related information may be placed in your permanent hospital, clinic, or physician’s office record;

1. **Your information may be given to:**

1. The U.S. Food and Drug Administration (FDA), Department of Health and Human Services (DHHS) agencies, and other federal and state entities;
2. Governmental agencies to whom certain diseases (reportable diseases) must be reported; and
3. The Ohio State University units involved in managing and approving the research study including the Office of Research and the Office of Responsible Research Practices.
4. **Why will this information be used and/or given to others?**

- To do the research;
- To study the results; and
- To make sure that the research was done right.

1. **When will my permission end?**

There is no date at which your permission ends. Your information will be used indefinitely. This is because the information used and created during the study may be analyzed for many years, and it is not possible to know when this will be complete.

1. **May I withdraw or revoke (cancel) my permission?**

Yes. Your authorization will be good for the time period indicated above unless you change your mind and revoke it in writing. You may withdraw or take away your permission to use and disclose your health information at any time. You do this by sending written notice to the researchers. If you withdraw your permission, you will not be able to stay in this study. When you withdraw your permission, no new health information identifying you will be gathered after that date. Information that has already been gathered may still be used and given to others.

1. **What if I decide not to give permission to use and give out my health information?**

Then you will not be able to be in this research study and receive research-related treatment. However, if you are being treated as a patient here, you will still be able to receive care.

1. **Is my health information protected after it has been given to others?**

There is a risk that your information will be given to others without your permission. Any information that is shared may no longer be protected by federal privacy rules.

1. **May I review or copy my information?**

Signing this authorization also means that you may not be able to see or copy your study-related information until the study is completed.

**16. Who can answer my questions about the study?**

For study questions, concerns, or complaints, to withdraw consent and HIPAA authorization, or if you feel you have been harmed as a result of study participation, you may contact:

Study Principal Investigator: [PI Name]

Email Address: [PI Email Address]

Phone number: [PI Phone Number]

For questions related to your privacy rights under HIPAA or related to this research authorization, please contact ­[privacyoffice@osumc.edu](mailto:privacyoffice@osumc.edu).

For questions about your rights as a participant in this study or to discuss other study-related concerns or complaints with someone who is not part of the research team, you may contact the Office of Responsible Research Practices at [Phone Number].

**(For virtual consent)** Once you receive the consent and authorization form through email or some other system (e.g., REDCap), you will have whatever time necessary to read it and complete if you wish. If you have questions or concerns, please contact [Study PI]

**Signing the consent form**

I have read (or someone has read to me) this form and I am aware that I am being asked to participate in a research study. I have had the opportunity to ask questions and have had them answered to my satisfaction. I voluntarily agree to participate in this study.

I am not giving up any legal rights by signing this form. I will be given a copy of this combined consent and HIPAA research authorization form**.**

|  |  |  | |
| --- | --- | --- | --- |
| **Printed name of participant** |  | **Signature of participant** | |
|  |  |  | **AM/PM** |
|  |  | **Date and time** |  |
|  |  |  |  |
|  |  |  | |
| **Printed name of person authorized to consent for participant (when applicable)** |  | **Signature of person authorized to consent for participant**  **(when applicable)** | |
|  |  |  | **AM/PM** |
| **Relationship to the participant** |  | **Date and time** |  |

**Investigator/Research Staff**

I have explained the research to the participant or his/her representative before requesting the signature(s) above. There are no blanks in this document. A copy of this form has been given to the participant or his/her representative.

|  |  |  | |
| --- | --- | --- | --- |
| **Printed name of person obtaining consent** |  | **Signature of person obtaining consent** | |
|  |  |  | **AM/PM** |
|  |  | **Date and time** |  |

**Witness(es) -** *May be left blank if not required by the IRB*

|  |  |  | |
| --- | --- | --- | --- |
| **Printed name of witness** |  | **Signature of witness** | |
|  |  |  | **AM/PM** |
|  |  | **Date and time** |  |
|  |  |  | |
| **Printed name of witness** |  | **Signature of witness** |  |
|  |  |  | **AM/PM** |
|  |  | **Date and time** |  |
