# Supplementary material for: Comparing the Accuracy of Different Wearable Activity Monitors in Patients With Lung Cancer and Providing Initial Recommendations: Protocol for a Pilot Validation Study
Source: JMIR Res Protoc. 2025 Jun 19;14:e70472. doi: 10.2196/70472 (PMC12226780; doi:10.2196/70472)

**RESEARCHER SOP: FITBIT DATA AUTHORIZATION WORKFLOW**

NOTE: You must be using the secured lab desktop computer to use the Fitbit Data Extraction tool

1. A lab-administered Fitbit account must be created for the patient. The account can be created at [www.fitbit.com](https://mcas-proxyweb.mcas.ms/certificate-checker?login=false&originalUrl=http%3A%2F%2Fwww.fitbit.com.mcas.ms%3FMcasTsid%3D20892&McasCSRF=62c2230ea795f3e70354b5551248de2d619914d6b6356113f9b8e8b8433282d1). For the username, enter the REDCAP Patient ID followed by “@gmail.com”. For the password, enter any randomly generated alphanumeric 10-character string

1. Prior to the initial patient visit, navigate to the following URL: [https://127.0.0.1:5000/.](https://127.0.0.1:5000/) Please note that the data authorization tool is only running locally on a single OSU administered secured desktop computer, and is only accessible from that computer

1. You will then see the following page. Click the "Add New User” (highlighted in red) button to begin the data authorization process


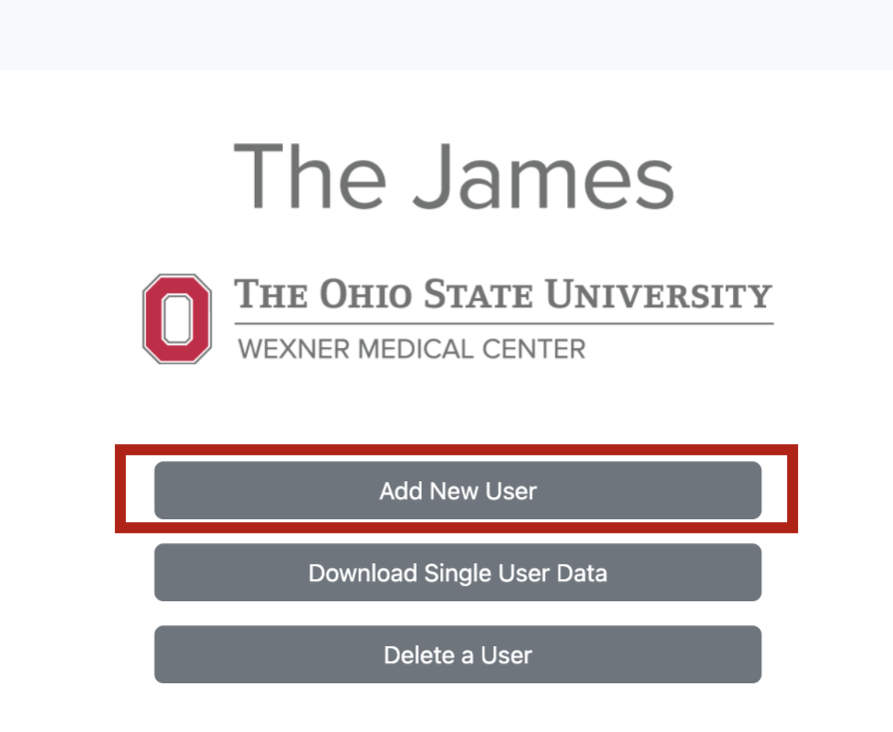


1. Click the “Login” button on the data extraction tool


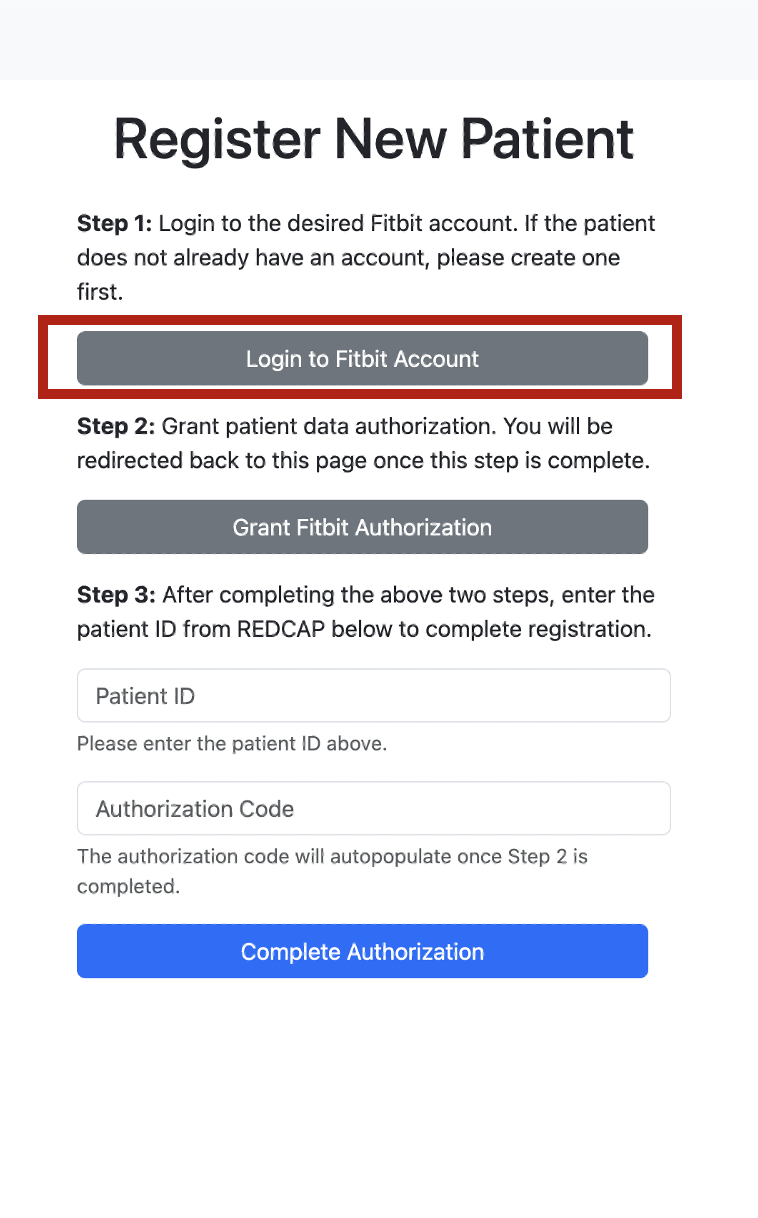


1. The application will then display the Fitbit login page

- Enter the username and password created in Step 1 and sign in


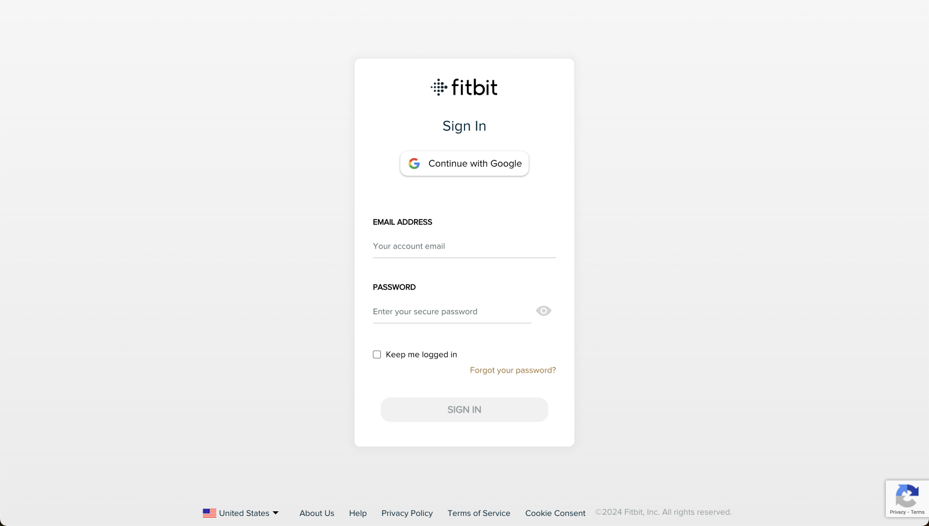


1. After signing in, close the Fitbit login tab and return to the *Fitbit Data Extraction Tool*. Click the “Grant Fitbit Authorization” button.


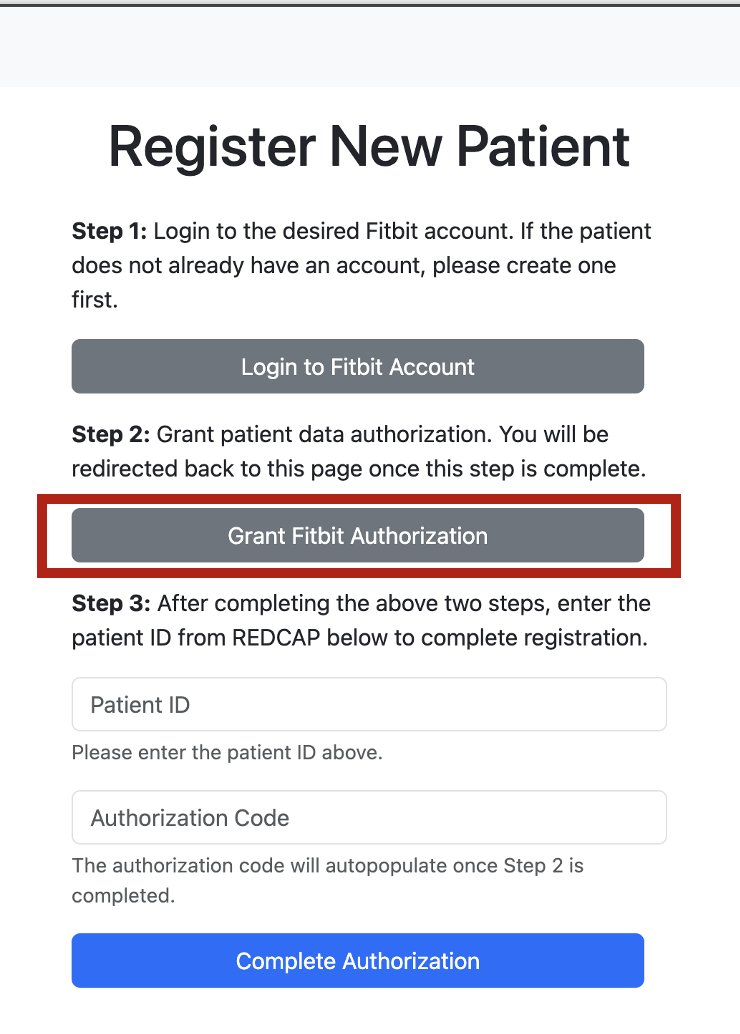


1. A popup will appear to enable data sharing of metrics. Select “weight, activity and exercise, sleep, heart rate, and Fitbit devices and settings” to grant access to data from the Fitbit device


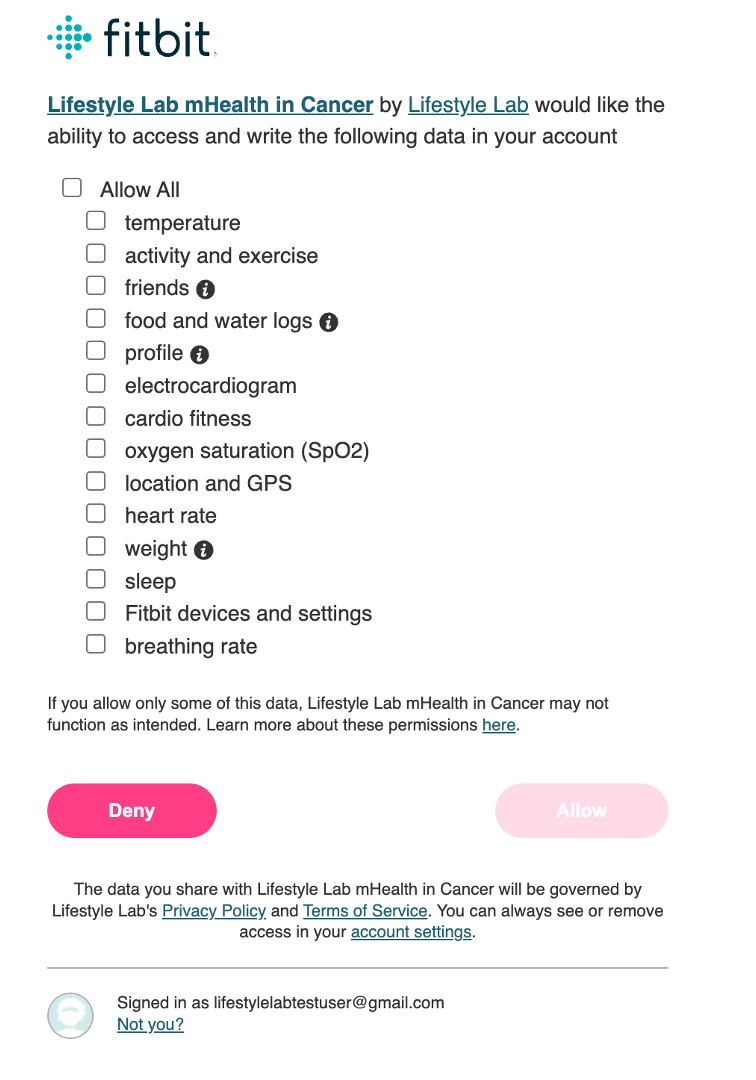


1. Once access has been granted, the application will redirect to the *Fitbit Data Extraction Tool*. Enter the Patient ID from REDCAP into the highlighted input field and click "Complete Authorization”.


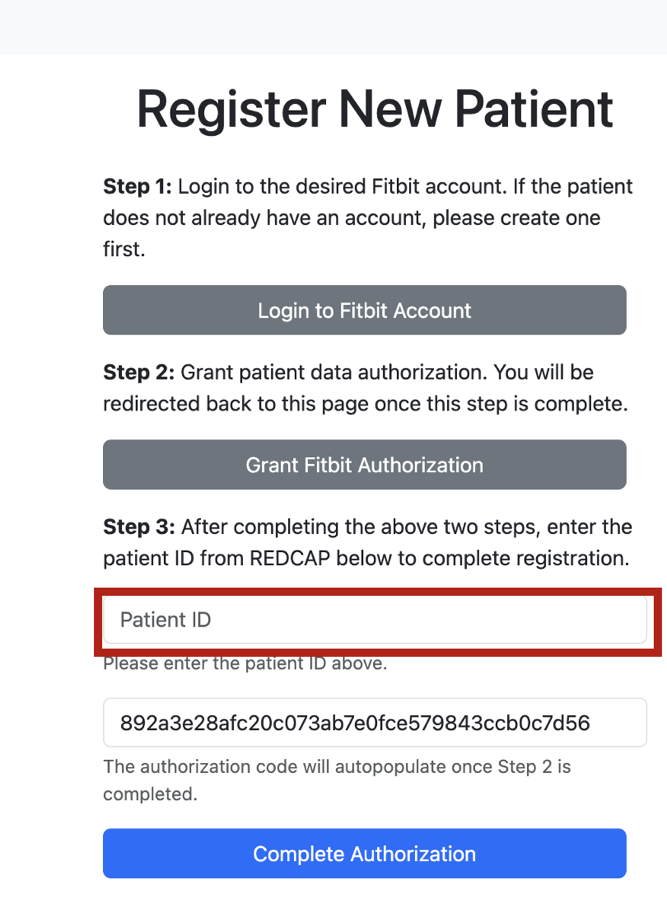


**RESEARCHER SOP: FITBIT DATA DOWNLOAD WORKFLOW**

- 1. Click the “Download Single User” button


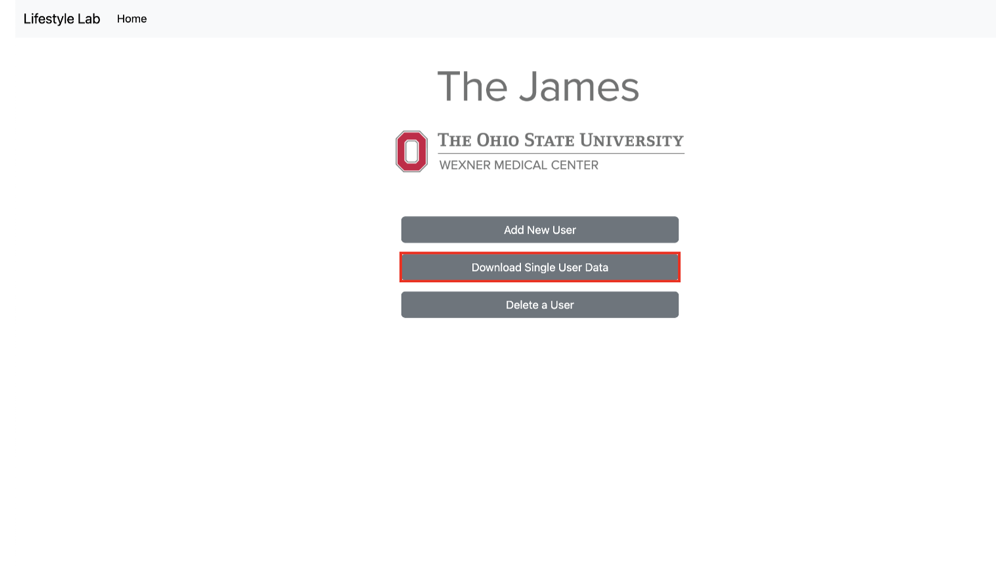


- 1. Select the user for whom you would like to download data, the first date of data collection, and the last date of data collection, and click on the “Download” button


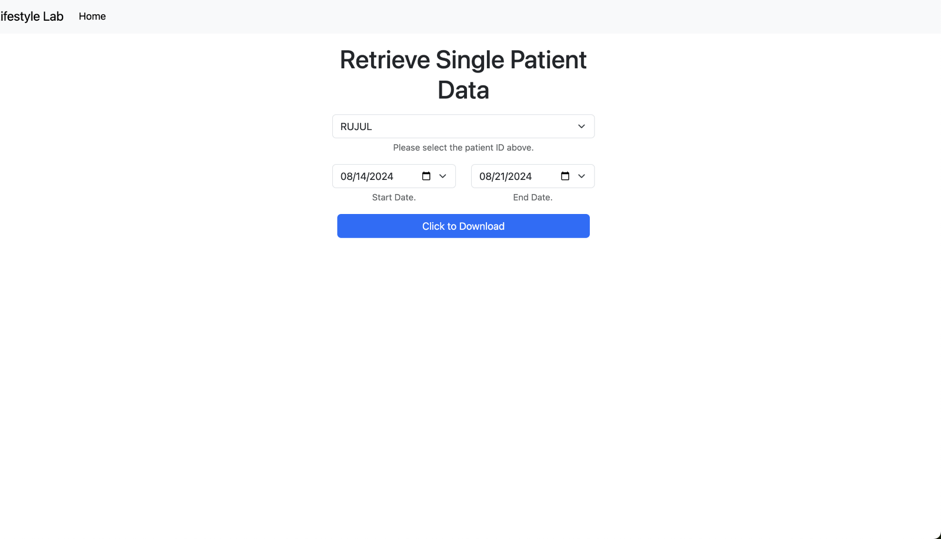

Supplement: Multimedia Appendix 4 [file resprot_v14i1e70472_app4.docx]
