# Supplementary material for: Comparing the Accuracy of Different Wearable Activity Monitors in Patients With Lung Cancer and Providing Initial Recommendations: Protocol for a Pilot Validation Study
Source: JMIR Res Protoc. 2025 Jun 19;14:e70472. doi: 10.2196/70472 (PMC12226780; doi:10.2196/70472)
Supplement: Multimedia Appendix 5 [file resprot_v14i1e70472_app5.docx]

**Activity Monitor Wear Instructions:**

- **Please wear the following** **devices for the entire 7-day period.**
- **Please do not lose these monitors. If you have lost any of these devices, please contact research staff immediately. (Contact information on the next page)**

**ActivPal**


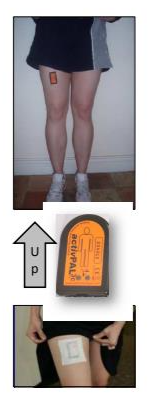


- To apply the monitor, please place the monitor in the middle of your right thigh with the curved edge facing you.. Use the provided Tegaderm film to secure the device to your thigh.
- Make sure to smooth the film down so that there are no air bubbles, and it is flush against your skin.
- **Please do not get the monitor wet, you may wear it while you shower but ensure that the Tegaderm is intact. Make sure to remove this device when you bathe/swimming, and log in the sleep/non-wear diary. You can keep the device on when showering**
- You may want to change the Tegaderm after 2 or 3 days.

**Fitbit Charge 6**


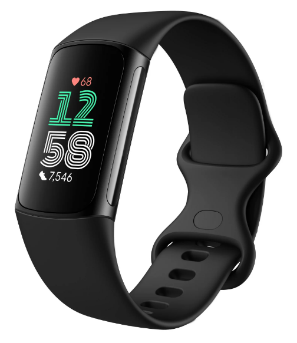


- Please wear on your non-dominant wrist.
- For everyday wear, tighten the band on your wrist, wear it loose enough so that it can move back and forth on your wrist.
- Please do not remove when you go to sleep.
- **Make sure to remove this device when you bathe/swim, and log in the sleep/non-wear diary. You can keep the device on when showering**
- During activity, please tighten the device so that it is snug on your wrist. Do not tighten so that it constricts blood flow, just enough so that it does not move around.
- If the device runs out of charge, place on the provided charger for 1-hour and log appropriately in the sleep/non-wear diary
- **If the app gives a firmware update notification, please ignore it and do not install the firmware update.**

**ActiGraph LEAP**


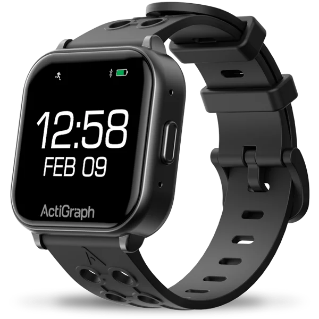


- Please wear on your non-dominant wrist.
- Tighten the band on your wrist so that it does not move around and there is no space between your wrist and the strap/device.
- Please do not remove when you go to sleep.
- **Make sure to remove this device when you bathe/swimming, and log in the sleep/non-wear diary. You can keep the device on when showering**
- During activity, please tighten the device so that it is snug on your wrist. Do not tighten so that it constricts blood flow, just enough so that it does not move around.
- If the device runs out of charge, place on the provided charger for 1-hour and log appropriately in the sleep/non-wear diary

**Research Contact Information:**

[Study PI Name and Contact Information]
