# Supplementary material for: Comparing the Accuracy of Different Wearable Activity Monitors in Patients With Lung Cancer and Providing Initial Recommendations: Protocol for a Pilot Validation Study
Source: JMIR Res Protoc. 2025 Jun 19;14:e70472. doi: 10.2196/70472 (PMC12226780; doi:10.2196/70472)
Supplement: Multimedia Appendix 6 [file resprot_v14i1e70472_app6.docx]

| **Today’s Date** | 1/26/24 |  |  |  |  |  |  |  |
| --- | --- | --- | --- | --- | --- | --- | --- | --- |
| **What time did you get into bed?** | 10:15 p.m. |  |  |  |  |  |  |  |
| **What time did you try to go to sleep?** | 10:30 p.m. |  |  |  |  |  |  |  |
| **How long did it take you to fall asleep?** | 1 hour |  |  |  |  |  |  |  |
| **How many times did you wake up, not counting your final awakening?** | 3 times |  |  |  |  |  |  |  |
| **In total, how long did these awakenings last?** | 1 hour  30 minutes |  |  |  |  |  |  |  |
| **What time was your final awakening?** | 6:30 a.m. |  |  |  |  |  |  |  |
| **What time did you get out of bed for the day?** | 6:45 a.m. |  |  |  |  |  |  |  |
| **How would you rate the quality of your sleep?**  **(please circle)** | Very poor  Poor  Fair  Good  Very good | Very poor  Poor  Fair  Good  Very good | Very poor  Poor  Fair  Good  Very good | Very poor  Poor  Fair  Good  Very good | Very poor  Poor  Fair  Good  Very good | Very poor  Poor  Fair  Good  Very good | Very poor  Poor  Fair  Good  Very good | Very poor  Poor  Fair  Good  Very good |
| **Did you take a nap today?** | Nap 1: 10-11am    Nap 2: 5:30-6pm |  |  |  |  |  |  |  |
| **Comments**  **(if applicable)** | I have a cold |  |  |  |  |  |  |  |
| **Did you take the monitor off at any time throughout the day/night?** | | | | | | | | |
| **Which monitor did you take off?** | ActiGraph |  |  |  |  |  |  |  |
| **Time off –**  **Time back on** | 2:00-2:45pm |  |  |  |  |  |  |  |
| **Reason(s) for taking off the monitor** | Swim class |  |  |  |  |  |  |  |

**Sleep Diary Instructions**

- Please fill out this form **every day**, ideally within 1 hour of when you wake up in the morning.
- You do not need to report exact times or watch the clock, you can make your best guess when answering these questions.
- Placing this sleep diary on a bedside table or at the table where you eat breakfast can help you remember to fill it out.

*Below are the questions included in the sleep log:*

1. **Today’s Date:**

- This is the date of the morning that you are filling the form out. Ex: Today is Tuesday the 11th so I put that date, but I am reporting times for Monday night’s sleep or the 10th.

1. **What time did you get into bed?**

- Write the time that you got into bed. If you got into bed at 10:15 but spent 10 minutes on your phone, you still got into bed at 10:15. If you got into bed and tried to fall asleep right away, this time may be the same time that you report for when you tried to go to sleep.

1. **What time did you try to go to sleep?**

- Write the time that you tried to go to sleep. This is the time that you closed your eyes, and this may be the same as the time you got into bed.

1. **How long did it take you to fall asleep?**

- Do your best to estimate how long it took you to fall asleep based on your answer to question 3. This does not need to be exact, please do not watch the clock, you can give your best guess for this question.

1. **How many times did you wake up, not counting your final awakening?**

- Write how many times you woke up between when you fell asleep and your final awakening.

1. **In total, how long did these awakenings last?**

- Write how long these awakenings lasted. If you woke up 2 times for 30 minutes and 45 minutes, add them together and report the total time (30 + 45 = 1 hour and 15 minutes).

1. **What time was your final awakening?**

- Write the time that you woke up for the final time in the morning. This may be the same time that you report for when you got out of bed. Usually, this is the time that your alarm clock went off, or when someone else woke you up, or when your body naturally woke up for the last time.

1. **What time did you get out of bed for the day?**

- Write the time that you got out of bed and started your day. This could be the same time that you woke up for the final time, depending on if you got out of bed right away or not.

1. **How would you rate the quality of your sleep?**

- Please circle which of the provided answers best represents how you slept.

1. **Comments (if applicable)**

- Write anything about your day or night that could’ve impacted your sleep duration or the quality of your sleep. Please note if you took any **naps**.

1. **Did you take the monitor off at any time throughout the day?**

- Write the time you took off the monitor and put it back on. Please provide a reason for taking off the monitor.
